# Genome-Wide Distribution of RNA-DNA Hybrids Identifies RNase H Targets in tRNA Genes, Retrotransposons and Mitochondria

**DOI:** 10.1371/journal.pgen.1004716

**Published:** 2014-10-30

**Authors:** Aziz El Hage, Shaun Webb, Alastair Kerr, David Tollervey

**Affiliations:** Wellcome Trust Centre for Cell Biology, University of Edinburgh, Edinburgh, United Kingdom; University of Michigan, United States of America

## Abstract

During transcription, the nascent RNA can invade the DNA template, forming extended RNA-DNA duplexes (R-loops). Here we employ ChIP-seq in strains expressing or lacking RNase H to map targets of RNase H activity throughout the budding yeast genome. In wild-type strains, R-loops were readily detected over the 35S rDNA region, transcribed by Pol I, and over the 5S rDNA, transcribed by Pol III. In strains lacking RNase H activity, R-loops were elevated over other Pol III genes, notably tRNAs, SCR1 and U6 snRNA, and were also associated with the cDNAs of endogenous TY1 retrotransposons, which showed increased rates of mobility to the 5′-flanking regions of tRNA genes. Unexpectedly, R-loops were also associated with mitochondrial genes in the absence of RNase H1, but not of RNase H2. Finally, R-loops were detected on actively transcribed protein-coding genes in the wild-type, particularly over the second exon of spliced ribosomal protein genes.

## Author Summary

R-loops (RNA-DNA hybrids) are potentially deleterious for gene expression and genome stability, but can be beneficial, for example, during immunoglobulin gene class-switch recombination. Here we made use of antibody S9.6, with specificity for RNA-DNA duplexes independently of their sequence. The genome-wide distribution of R-loops in wild-type yeast showed association with the highly transcribed ribosomal DNA, and protein-coding genes, particularly the second exon of spliced genes. On RNA polymerase III loci such as the highly transcribed transfer RNA genes (tRNAs), R-loop accumulation was strongly detected in the absence of both ribonucleases H1 and H2 (RNase H1 and H2), indicating that R-loops are inherently formed but rapidly cleared by RNase H. Importantly, stable R-loops lead to reduced synthesis of tRNA precursors in mutants lacking RNase H and DNA topoisomerase activities. RNA-DNA hybrids associated with TY1 cDNA retrotransposition intermediates were elevated in the absence of RNase H, and this was accompanied by increased retrotransposition, in particular to 5′-flanking regions of tRNAs. Our findings show that RNase H participates in silencing of TY1 life cycle. Surprisingly, R-loops associated with mitochondrial transcription units were suppressed specifically by RNase H1. These findings have potentially important implications for understanding human diseases caused by mutations in RNase H.

## Introduction

During transcription, the RNA polymerase opens the DNA duplex, and in the process rotates the DNA double helix by approximately one turn per 10 bp. This generates positive torsional stress ahead, and negative torsional stress in the wake, of the transcribing polymerase [1]. Positive stress impedes further unwinding of the DNA duplex, potentially stalling the polymerase. In contrast, negative torsion can lead to DNA strand separation and opening of the duplex. The resulting template single-stranded DNA region can base-pair with the nascent RNA transcript, generating an RNA-DNA duplex and an unpaired non-template DNA strand, giving rise to the term “R-loop” for such structures (for reviews see [2], [3], [4], [5], [6], [7], [8]).

Other features besides negative topological stress strongly influence R-loop formation [3], e.g. the G.C content of the inherent sequence. In particular, R-loop formation can be favoured by a high guanine (G) density in the non-template DNA strand (property known as positive GC skew, see [9], [10]), and this is specifically due to the higher thermodynamic stability of RNA-DNA hybrid sequences endowed with “G-rich purine RNA”/“C-rich pyrimidine DNA” duplexes [9], [10], [11], [12], [13], [14]. Importantly, R-loops rich in G-clusters have been linked to immunoglobulin class switch recombination and CpG methylation in mammals [9], [10], [14], [15].

R-loops are generally regarded as highly deleterious, since the single stranded DNA is susceptible to damage. Moreover, it is believed that the structure can block both transcription and DNA replication, creating replicative stress and potentially causing further DNA damage (for reviews see [2], [3], [5], [6], [7]. Highly transcribed genes in yeast exhibit greater mutation and recombination rates than genes transcribed at lower rates (reviewed in [7]), which might be related to R-loop formation.

R-loops can be resolved by RNase H1 and/or RNase H2 (Rnh201 is the catalytic subunit of a three subunit enzyme), either of which can cleave the RNA component in the RNA-DNA hybrid, albeit with different efficiencies (reviewed in [16]). However, loss of both RNase H1 and H2 activity is not lethal in yeast [17], strongly indicating that other cellular activities can resolve R-loops, such as the helicase Sen1/Senataxin, THO/TREX RNA packaging complexes and the RNA exosome [2], [5], [8]. Moreover, RNase H2 plays dual roles in preserving genome integrity, processing both R-loops and ribonucleotides mis-incorporated in to DNA during replication, whereas RNase H1 is reported to resolve only R-loops (reviewed in [16], [18]). In mammals both RNase H1 and H2 are required for cell viability and for embryonic development, and mutations in any of the three subunits of RNase H2 have been reported to cause the neuro-inflammatory disease Aicardi-Goutières syndrome (AGS) [19], [20], [21], [22].

In previous analyses of transcription by RNA polymerase I (Pol I) on the yeast ribosomal DNA (rDNA), we observed that R-loops are common at specific sites, in particular within the 5′-region of the 18S rDNA [23]. These were readily detected in wild-type strains, although their abundance was increased in strains lacking the activity of DNA Topoisomerase I (Top1), which can resolve negative torsion behind the RNA polymerase (for a review on DNA topoisomerases see [24]), and further increased in the absence of RNase H activity. R-loop formation by Pol I on the highly transcribed rDNA array is favored by negative torsional stress [23], [25], suggesting the possibility that R-loop formation in wild-type cells might also be associated with other RNA polymerases, particularly on actively transcribed genes.

Here we determined the genome-wide distribution of RNA-DNA hybrids in budding yeast using chromatin immunoprecipitation (ChIP) with antibody S9.6 [26], [27], followed by deep sequencing of immunopurified DNA fragments (ChIP-seq). The conclusions are related to, but not identical with, the results of recent microarray analyses [11]. R-loops were strongly associated with actively transcribed loci by all RNA polymerases including the mitochondrial RNA polymerase (mtRNAP). Notably, R-loops accumulated unevenly across intron-containing genes with the highest peak over exon 2. We show that R-loop accumulation at tRNA genes leads to reduced pre-tRNA synthesis specifically in mutants lacking both RNase H and Top1, or also Top2 activities. We also show that integration of TY1 retrotransposons in 5′-flanking regions of tDNAs is favored in cells depleted of both Top1 and cellular RNase H activities. We present evidence that both RNase H1 and RNase H2 are involved in cleaving RNA-DNA hybrids associated with cDNAs of TY1 retrotransposons. Unexpectedly, we also show that only RNase H1 is involved in processing of co-transcriptional R-loops at mtDNA transcription units.

## Results

### Genome-wide mapping of R-loop locations by ChIP-seq

We previously reported that R-loops can be identified robustly by ChIP-QPCR analyses using the S9.6 antibody [23], which is specific for the structure of RNA-DNA duplexes independently of their sequence [26], [27]. To assess the genome-wide distribution of R-loops, formaldehyde-crosslinked and sonicated chromatin was incubated with antibody S9.6. The immunoprecipitated DNA and input chromatin were processed for high-throughput sequencing. Recovered DNA sequences were then mapped to the yeast genomic sequence (GEO Series accession number GSE53420).

**Figure 1:**
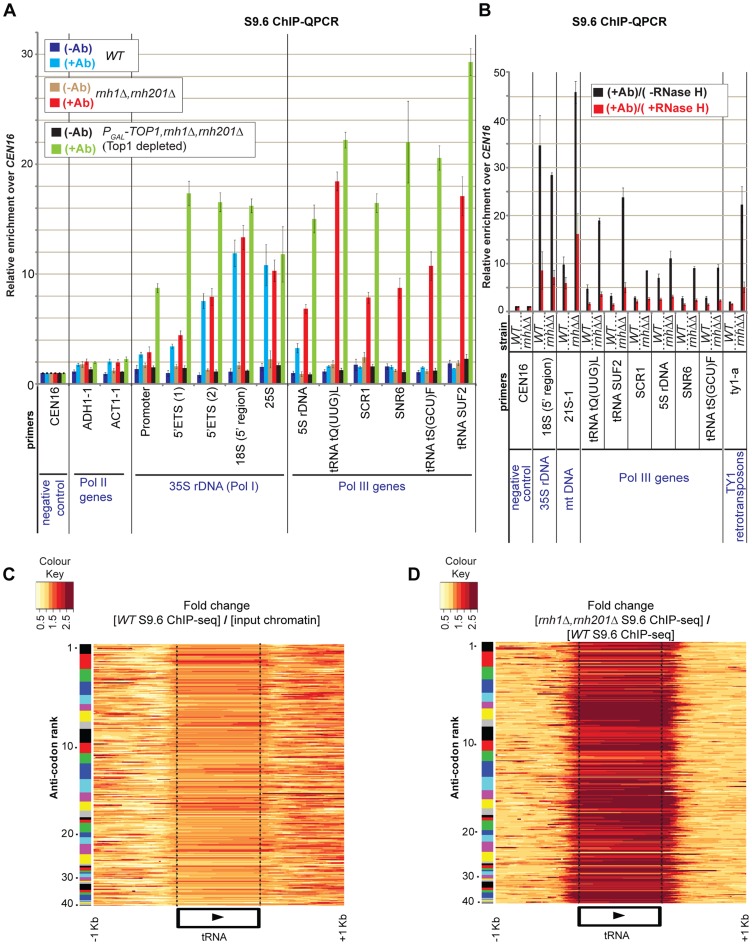
R-loops generated by RNA Polymerase III are substrates for cellular RNase H. **A**: Analysis of R-loops by ChIP-QPCR using antibody S9.6 in wild-type strain BY4741 (WT) and double mutant *rnh1*Δ *rnh201*Δ, and in triple mutant *P_GAL_-TOP1 rnh1*Δ *rnh201*Δ depleted of Top1 for 6h at 30°C. *CEN16*, the Pol II genes *ADH1* and *ACT1*, the Pol I transcribed 35S rRNA gene, and the Pol III genes (5S rDNA, tRNA *tQ(UUG)L*, *SCR1*, *SNR6*, tRNA *tS(GCU)F* and tRNA *SUF2*) were analyzed by Q-PCR. Values for no-antibody (−Ab) and antibody S9.6 (+Ab) were calculated as described in Materials and Methods and normalized to *CEN16*, which was set arbitrarily to 1 in order to compensate for differences in immunoprecipitation efficiencies. *CEN16* is not expected to be transcribed and should therefore give rise only to background signal after immunoprecipitation. The mean of three independent experiments is shown with standard error. **B**: S9.6-ChIP samples of strains WT (BY4741) and double mutant *rnh1*Δ *rnh201*Δ, grown at 30°C in YEPD (glucose 2%), were treated or not with recombinant RNase H ‘on-beads’ for 2.5 h at 37°C. *CEN16*, 18S rDNA, mitochondrial 21S rDNA, Pol III genes [same as in (A)] and Ty1 retrotransposons, were analyzed by Q-PCR as described above. *rnh*ΔΔ = double mutant *rnh1*Δ *rnh201*Δ. **C–D**: Heatmaps of R-loop distribution across 274 tRNA genes assigned to 41 families of distinct codon specificity in the WT (C) and double mutant *rnh1*Δ *rnh201*Δ (D). Genes are ordered by their anticodon rank (Y-axis) based on the sum of codons in the genome for which it can be used, with a value of 1 representing the anticodon with the highest number of codons in the genome. Anticodons were grouped into 41 families (see [99]), and the codon frequencies were calculated from all protein coding genes in the Saccer3 genome assembly. The X-axis shows the position of the tRNA genes with 1 kb of 5′- and 3′- flanking sequences. Each point on the graph is a colored tile representing the fold change of: “[WT S9.6 ChIP-seq] relative to [input chromatin]” panel (C); and “[*rnh1*Δ *rnh201*Δ S9.6 ChIP-seq] relative to [WT S9.6 ChIP-seq]” panel (D). 5′ and 3′ endpoints of mature tRNAs are delineated by vertical dotted lines across the heatmaps.

ChIP-seq was applied to wild-type and to mutants double *rnh1*Δ *rnh201*Δ and triple *P_GAL_::TOP1 rnh1*Δ *rnh201*Δ (Fig. S1). For Top1 depletion, cells were shifted from medium containing galactose plus sucrose and harvested after 6 h in glucose-containing medium. Sequenced reads over each target were normalized to the genome-wide mean of all intergenic regions (arbitrarily set as sequencing background, see Materials and Methods), so changes in hit densities are relative differences compared to all other targets. Reads mapped to the rDNA in strains WT, *rnh1*Δ *rnh201*Δ and *P_GAL_::TOP1 rnh1*Δ *rnh201*Δ (depleted of Top1), were greatly enriched in the S9.6 ChIP-seq data over the input chromatin (Fig. S1B). This is a good indication that R-loops are strongly associated with this locus, as also observed in S9.6 ChIP-QPCR (Figs. 1A–B; and [23]). For the Pol I transcribed, 35S pre-rRNA region of the rDNA, the strongest peak detected in the wild-type strain was located over the 5′ segment of the 18S rDNA (region ∼210 nt to ∼580 nt at the beginning of 18S rRNA; triple asterisk in Fig. S1A). Additional peaks were located over the 25S rDNA (e.g. quadruple asterisk in Fig. S1A). In strains lacking both cellular RNase H and Top1 a new peak appeared over the Pol I promoter and 5′ETS regions at the 5′ end of the 35S pre-rRNA (double asterisk in Fig. S1A; see also ChIP-QPCR in Fig. 1A). A further prominent peak was seen over the 5S rDNA, which is transcribed by RNA Pol III in the opposite direction to the 35S pre-rRNA (single asterisk in Fig. S1A). Notably, R-loops over the 5S rDNA were strongly increased in strains lacking RNase H and even more when Top1 was also absent (single asterisk in Fig. S1A; see also ChIP-QPCR in Fig. 1A). Comparison to DNA base-composition indicated that the uneven distribution of R-loops over the transcribed regions of the rDNA partially reflects a preference for C+G rich sequences (Fig. S1A).

To confirm that the signals detected by the antibody represent bona fide sites of RNA-DNA hybrids, wild-type ChIP samples were treated on-beads with recombinant *E. coli* RNase HI *in vitro* followed by recovery and analysis of the bound DNA (Fig. 1B). A strong reduction in the R-loop signal was seen over the rDNA region in the RNase H treated samples.

### Pol III genes are targets for RNase H activity

ChIP-QPCR for the 5S rRNA, the cytoplasmic RNA scR1, the small nuclear RNA U6 (SNR6) and three tRNA genes (Fig. 1A) revealed that R-loops are also associated with these loci. In the wild-type strain R-loops are detected at the 5S rDNA (compare dark blue +Ab and light blue −Ab bars), as also observed for the 35S rDNA. However, for other Pol III transcripts R-loops were strongly increased by the absence of RNase H activity (*rnh1*Δ *rnh201*Δ, red +Ab bars). In each case, R-loop accumulation was further increased by depletion of Top1, although this increase was relatively small in the case of tRNA genes (*P_GAL_-TOP1 rnh1*Δ *rnh201*Δ, light green +Ab bars). *In vitro* treatment of S9.6 ChIP samples with recombinant RNase H strongly reduced R-loops associated with Pol III genes (Fig. 1B). This confirms that Pol III fragments, which are immunoprecipitated by antibody S9.6 indeed represent sites of RNA-DNA hybrid formation.

The conclusion that R-loop accumulation is highly increased over tRNA genes in strains lacking RNase H activities was supported by ChIP-Seq data (Figs. 1C–D). The expression levels of tRNA genes are correlated with the usage of the corresponding codon in mRNAs, but this is offset by the greater numbers of genes encoding isoacceptors for the most common codons [28], [29], [30]. Separation of the tRNAs into 41 gene families, ranked by codon usage, indicated that R-loop occupancy is heterogeneous between isogenic tRNAs of each family (Figs. 1C–D; number 1 indicates the most common anticodon). Importantly also, some tRNA genes were enriched over their entire transcribed region, some showed higher levels of enrichment over their 5′ region, and some other showed enrichment over their 3′ region. This heterogeneity in R-loop occupancy between and within tRNA genes is likely to reflect heterogeneity in tRNA transcription rates among isoacceptors [28], [29], [30], as well as differences in the thermodynamic stability of the RNA-DNA hybrids [12], [13]. It should be noted that the fold enrichment of R-loops at tRNA genes in strains lacking RNase H (or also Top1) relative to input chromatin (or to the WT) in ChIP-Seq data were generally lower than those in ChIP-QPCR data (compare Figs. 1C–D with 1A). This may reflect differences in ChIP-seq efficiencies within and between samples.

For tRNA heatmaps in Figs. 1C–D, only sequence reads mapping to unique locations on the genome were used, thus excluding the possibility that hits at one tRNA isogene could be erroneously attributed to other family members. Notably, the distribution of hits extends beyond the ends of the mature tRNA species into the non-conserved flanking regions (indicated by dotted lines in Figs. 1C–D), confirming that the recovered sequences are unique and arise from the genomic loci. The R-loops overlapping 5′ and 3′ flanking regions of mature tRNA species potentially play roles in initiation and termination of transcription by Pol III ([31], [32]).

The substantial increase in R-loops on tRNA genes seen in strains lacking RNase H activity presumably reflects their high transcription rates, whereas low levels of R-loops in wild-type strains apparently shows that these are normally cleared rapidly by RNase H. However, the ChIP-QPCR data (Fig. 1A) revealed little further increase in strains also depleted of Top1. We speculate that due to their generally short lengths, Pol III genes are less dependent on topoisomerase activity than are the long Pol I transcripts [23], [25], [33].

### Alterations in pre-tRNA metabolism in strains with increased R-loops at tRNA genes

The presence of R-loops is expected to impede transcription elongation, increasing the time required for pre-tRNA synthesis. In contrast, the accumulation of negative supercoiling behind the polymerase in strains with reduced topoisomerase activity can increase transcription initiation rates via promoter opening.

**Figure 2:**
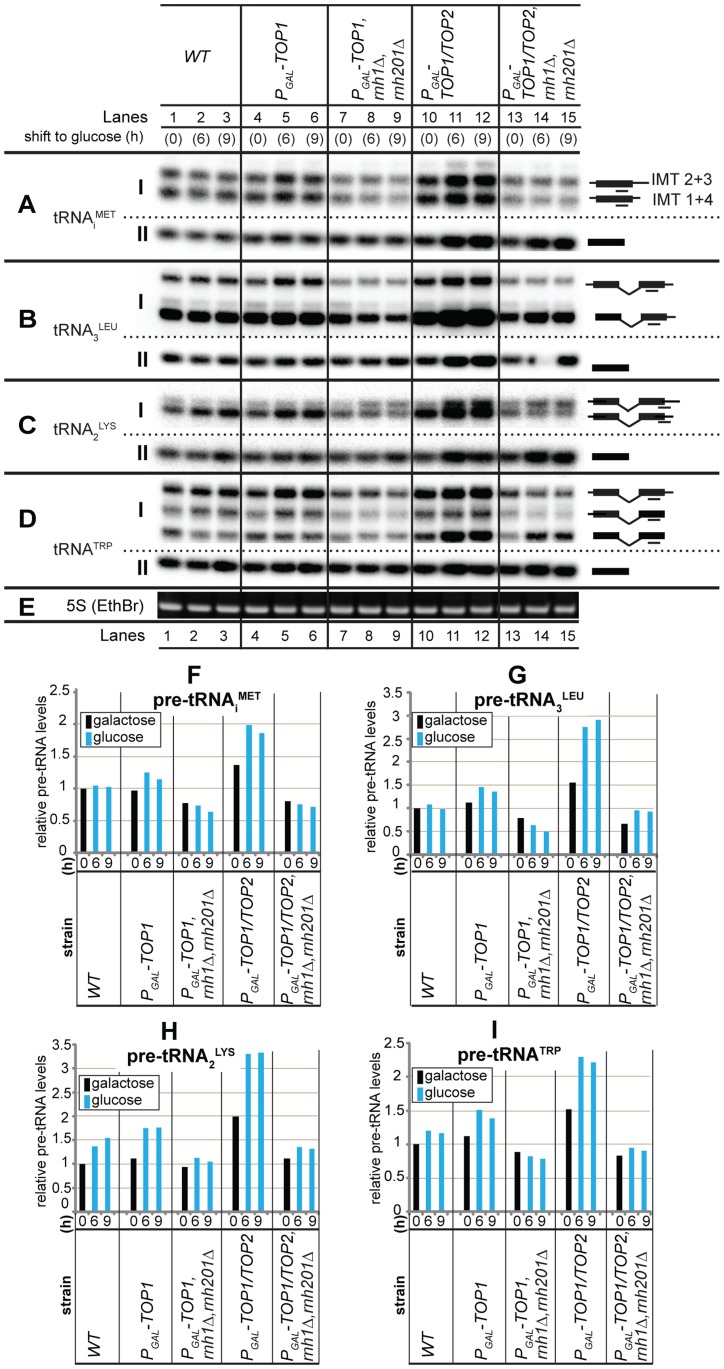
R-loops at tRNA genes affect pre-tRNA synthesis in strains lacking topoisomerase and RNase H activities. **A–E**: Wild-type strain BY4741 (*WT*) and isogenic mutant strains single *P_GAL_-TOP1*, triple *P_GAL_-TOP1 rnh1*Δ *rnh201*Δ, double *P_GAL_-TOP1/TOP2* and quadruple *P_GAL_-TOP1/TOP2 rnh1*Δ *rnh201*Δ, were grown at 30°C and harvested at 0 h (galactose- and sucrose- containing medium) and at 6–9 h post-shift to glucose-containing medium. Total RNAs were extracted and analyzed by northern hybridization. The membrane was hybridized separately with probes tRNA_i_^MET^ (A), tRNA_3_^LEU^ (B), tRNA_2_^LYS^ (C) and tRNA^TRP^ (D). Ethidium bromide staining of 5S rRNA is in (E). Precursor and mature tRNA species are in subpanels I and II, respectively. Exon and intron sequences are represented by filled boxes and horizontal lines, respectively, and probe locations are indicated as lines under the schematics of the pre-tRNA species. Short (*IMT1*+*IMT4*) and long (*IMT2*+*IMT3*) forms of tRNA_i_^MET^ are indicated. **F–I**: Quantification of tRNA precursors (pre-tRNAs) from northern analysis data in panels A–D. Pre-tRNA ratios at each time point were generated by normalising the pre-tRNA species (subpanels I) to the loading control (5S rRNA) and by expressing all the values relative to the 0 h sample of the wild-type strain, which was set to 1.

To assess the outcome of these potentially competing effects, pre-tRNA levels were assessed in strains genetically depleted of Top1 (single *P_GAL_-TOP1*), or of both Top1 and Top2 (double *P_GAL_-TOP1 P_GAL_-TOP2*, designated *P_GAL_-TOP1/TOP2* in Fig. 2), or also lacking RNase H activity (triple *P_GAL_-TOP1 rnh1*Δ *rnh201*Δ and quadruple *P_GAL_-TOP1 P_GAL_-TOP2 rnh1*Δ *rnh201*Δ). Pre-tRNA synthesis was slightly affected under conditions of partial induction of *P_GAL_-TOP1*, specifically at 0 h depletion in medium containing galactose plus sucrose (which provides the cells with limited amounts of glucose), in both the triple and quadruple mutant strains, relative to the strains with functional RNase H (Figs. 2A–D panels I, compare lanes 7 and 13 with 1, 4 and 10; quantified in Figs. 2F–I).

Following a shift to repressive, glucose-containing media, Top1 depletion in the single mutant resulted in elevated pre-tRNA levels, and this was further increased when Top2 was also depleted in the double mutant (Figs. 2A–D, panels I, lanes 5–6 and 11–12; quantified in Figs. 2F–I). Elevated levels were seen for the unprocessed primary transcripts and the unspliced but end-matured pre-tRNAs (Figs. 2B–D, lanes 5–6 and 11–12, panels I). Similarly, elevated levels were also seen for the intronless pre-tRNA species (Fig. 2A, lanes 5–6 and 11–12, panel I). However, loss of RNase H activity reversed this accumulation in each case. Indeed, the triple and quadruple mutant strains had a reduced ratio of precursor to mature tRNA for several species tested at 0 h and 6–9 h post-shift to glucose-containing medium (Figs. 2A–D, lanes 7–9 and 13–15, compare panels I and II).

Changes in transcription elongation rates are reported to impact on pre-mRNA and pre-rRNA maturation pathways (reviewed in [34], [35]), and our data indicate that this may also be the case for pre-tRNAs.

### Integration of TY1 retrotransposons at tDNAs is favored when Top1 and RNase H are lacking

**Figure 3:**
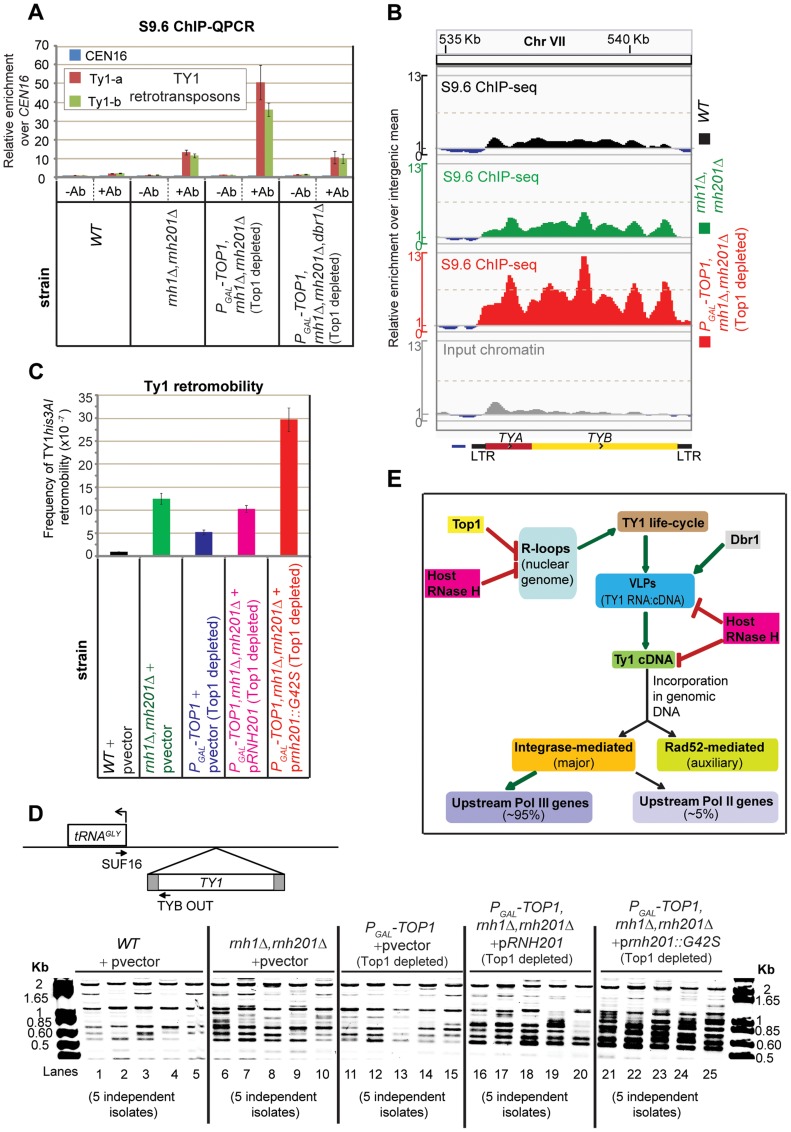
Cellular RNase H suppresses the mobility of Ty1 LTR-retrotransposons. A: Ty1 elements were analyzed by ChIP-QPCR for distribution of RNA-DNA hybrids in *WT* (BY4741) and double mutant *rnh1*Δ *rnh201*Δ, and in triple mutant *P_GAL_-TOP1 rnh1*Δ *rnh201*Δ and quadruple mutant *P_GAL_-TOP1 rnh1*Δ *rnh201*Δ *dbr1*Δ depleted of Top1 for 6 h at 30°C. ChIP samples and normalization of Q-PCR values to *CEN16* are as in Fig. 1A. The mean of three independent experiments is shown with standard error (two independent experiments for the quadruple mutant). Ab = antibody S9.6. B: S9.6 ChIP-seq profiles over the Ty1 retrotransposon *YGRWTY1-1* in the *WT* (BY4741), double mutant *rnh1*Δ *rnh201*Δ, and triple mutant *P_GAL_-TOP1 rnh1*Δ *rnh201*Δ depleted of Top1 for 61h at 30°C. Input chromatin is shown for the *WT*. Shown below is a graphical representation of a Ty1 element, which is comprised of *TYA* and *TYB* open reading frames flanked by long terminal repeats (LTR). The direction of Pol II transcription is indicated by arrowheads. The y-axis represents the relative enrichment of reads where values>1 are above the background level of sequencing (i.e. general intergenic mean, see Materials and Methods). Profiles were generated using the Integrative Genomics Viewer [100]. C: Bar diagrams showing the frequencies of Ty1*his3AI* mobility after complementation of the wild-type JC3212 (BY4741 *TY1his3AI-[Δ1]-3114*, [41]) and the mutants double *rnh1*Δ *rnh201*Δ and single *P_GAL_-TOP1* with a vector control, and the triple mutant *P_GAL_-TOP1 rnh1*Δ *rnh201*Δ with a vector expressing either wild-type Rnh201 or AGS-related mutant Rnh201_G42S_ [42]. Strains were grown until saturation at 18°C (for growth conditions see Materials and Methods). The frequency of *Ty1his3AI* mobility is the number of His+ prototrophs divided by the total number of cells plated (see Materials and Methods). The mean of two independent experiments of five independent isolates for each of the strains is shown with standard error. **D**: PCR analyses showing the integration of Ty1 at the 16 tRNA^GLY^ genes. *Upper panel*. Graphical representation of the integrated *Ty1* element at 5′-flanks of tRNA^GLY^ loci. Primers TYBOUT and SUF16 complementary to *Ty1* element and tRNA^GLY^ respectively were used for PCR amplification. *Lower panel*. Example of SYBR-stained gel showing integration of Ty1 cDNA upstream of any of the 16 tRNA^GLY^ gene loci. Five independent isolates were tested for each strain. Flanking lanes show DNA ladders with lengths in base-pairs (bp). The same yeast cultures served for both analyses in (C) and (D). E: Model for the role of co-transcriptional R-loops in activation of Ty1 retrotransposition (see Discussion). VLP = viral-like particle. Red thick arrow = negative regulation. Green thick arrow = positive regulation. For a detailed review on the mechanisms of TY1 retrotransposition see [36], [37]. See also model in Fig. S7.

Ty1 LTR-retrotransposons are composed of 2 direct long terminal repeats (LTRs) flanking the *TYA* and *TYB* open reading frames (see Fig. 3B; and [36], [37]). *TYA* encodes the Gag structural proteins of the virus-like particle (VLP), whereas *TYB* encodes the protease, the integrase and the reverse-transcriptase/RNase H (RT/RNase H). ChIP-QPCR analyses revealed only low levels of RNA-DNA hybrids over Ty1 retrotransposons in the wild-type strain, but notable accumulation was seen in the double mutant *rnh1*Δ *rnh201*Δ, and even more in the triple mutant *P_GAL_-TOP1 rnh1*Δ *rnh201*Δ following depletion of Top1 for 6 h (Fig. 3A). S9.6 ChIP-seq profiles showed that RNA-DNA hybrids are unevenly enriched across the Ty1 elements in the RNase H mutants (Figs. 3B and S2). *In vitro* treatment of S9.6 ChIP samples of double mutant *rnh1*Δ *rnh201*Δ with recombinant RNase H strongly reduced the signals over Ty1 retrotransposons confirming thus that these elements are associated with RNA-DNA prone sites (Fig. 1B).

The life-cycle of Ty1 involves transcription of a chromosomal element by Pol II, reverse transcription of Ty1 genomic mRNA into cDNA by the RT/RNase H inside the VLPs and incorporation of the cDNA into the nuclear genome (reviewed in [36], [37]). This raised the question of whether RNA-DNA hybrids mapped to Ty1 elements in the absence of cellular RNase H enzymes are produced by Pol II co-transcriptionally on the chromosomal elements (R-loops) or by reverse transcription in the VLPs (Ty1cDNA::RNA hybrid molecules).

Transposition of endogenous Ty1 elements is most frequent during growth at 22°C and below, much less active at 30°C and undetectable at 37°C [38]. Ty1 cDNA levels were quantified by Southern analysis of PvuII digested, total DNA. This showed an ∼3 fold increase in cDNA molecules in the double *rnh1*Δ *rnh201*Δ strain relative to the wild-type, in cultures grown at 22°C (Fig. S3B). To directly assess the role of RT in generating RNA-DNA duplexes on TY1, cultures of double *rnh1*Δ *rnh201*Δ strains were treated with the RT inhibitor phosfonoformic acid (PFA) (e.g. see [39]). This greatly reduced the accumulation of RNA-DNA hybrids at TY1 (see S9.6 ChIP-QPCR in Fig. S4), but not at other sites. This indicates that RNA-DNA hybrids over TY1 retrotransposons in *rnh1*Δ *rnh201*Δ strains are mostly associated with Ty1 cDNA molecules.

During growth at 30°C, Ty1 cDNA accumulated in the triple *P_GAL_-TOP1 rnh1*Δ *rnh201*Δ mutant following 6 h depletion of Top1 (Fig. S3C; compare lane 12 with 4). Loss of the intron-lariat debranching enzyme Dbr1 was reported to silence Ty1 retrotransposition, possibly by suppressing Ty1 replication in the VLPs [40]. The basis of this effect is unclear, but may be a consequence of the accumulation of high levels of intron lariats. The triple *P_GAL_-TOP1 rnh1*Δ *rnh201*Δ and quadruple *P_GAL_-TOP1 rnh1Δ rnh201Δ dbr1Δ* strains were compared following 6 h depletion of Top1 at 30°C. Loss of Dbr1 reduced the accumulation of RNA-DNA hybrids at Ty1 elements in ChIP-QPCR analyses (Fig. 3A), and Ty1 cDNA in Southern analysis (Fig. S3C, compare lanes 12 and 16). Western blotting showed that the abundance of the Gag protein p45 was slightly increased in the *P_GAL_-TOP1 rnh1*Δ *rnh201*Δ strain depleted of Top1, relative to the wild-type, at 30°C (Fig. S5). Together these data indicate that most RNA-DNA hybrids mapped to Ty1 elements in the absence of RNase H are associated with TY1 mRNAs, undoubtedly arising during reverse transcription of these into cDNAs in the VLPs (see model in Fig. 3E). However, we cannot exclude the possibility that some of these RNA-DNA hybrids are co-transcriptional R-loops generated by Pol II transcription of chromosomal Ty1 loci, as recently proposed [11].

To assess the effects of loss of RNase H and Top1 on endogenous Ty1 retromobility, we used a BY4741 strain carrying a *his3(AI)* construct inserted into a chromosomal Ty1 element (*TY1his3AI-[Δ1]-3114*, see ) [41]. The *his3(AI)* gene does not produce functional HIS3 mRNA, however, an intact *HIS3* gene can be regenerated by splicing, cDNA synthesis and retrotransposition. Ty1 mobility can therefore be quantified by measuring the rate of His+ prototroph formation. In strains carrying double *rnh1*Δ *rnh201*Δ or single *top1Δ* mutations, the rates of *TY1his3AI* transposition were ∼12.5 and ∼5 fold higher, respectively, than the isogenic wild-type (Fig. 3C). Yeast strains are unable to grow when all three enzymes Top1, RNase H1, and RNase H201 are absent [23]. We therefore complemented the triple mutant *P_GAL_-TOP1 rnh1*Δ *rnh201*Δ by expression of the AGS-related mutant protein Rnh201_G42S_ that shows reduced cleavage activity of RNA-DNA hybrids [42]. Ty1 mobility in this strain was ∼30 fold higher than in the wild-type and ∼3 fold greater than the *rnh1*Δ *rnh201*Δ strain (Fig. 3C). These data show that RNase H and Top1 act together to suppress endogenous Ty1 retromobility (see model in Fig. 3E).

Ty1 preferentially integrates in a ∼1 kb window upstream of Pol III-transcribed genes, at the nucleosomal H2A/H2B interface, with an approximate 80-bp periodicity between integration hotspots [43], [44]. To determine whether Top1 and/or RNase H play roles in the targeting of endogenous Ty1 to the 5′-flanking sequences of tRNA genes, we made use of a qualitative PCR assay (Fig. 3D). This yields a PCR product whenever a TY1 element integrates upstream from any of the 16 different tRNA^GLY^ genes. Analysis of DNA samples from the cultures used in Fig. 3C showed a large increase in Ty1 integration upstream of tRNA^GLY^ in the strain *P_GAL_-TOP1 rnh1*Δ *rnh201*Δ, depleted of Top1 and expressing the AGS-related protein Rnh201_G42S_ (Fig. 3D, lanes 21–25), relative to the wild-type (Fig. 3D, lanes 1–5). However, there was only a small increase in Ty1 integration in the double *rnh1*Δ *rnh201*Δ mutant (Fig. 3D, lanes 6–10; see also Fig. S6, odd numbered lanes, *rnh1*Δ *rnh201*Δ mutants). Integration of Ty1 at tRNA^GLY^ was strongly suppressed in strains lacking only Dbr1 or both Dbr1 and RNase H, relative to the loss of only RNase H (Fig. S6, even numbered lanes). We conclude that Top1 and RNase H act together to restrict Ty1 integration at sites 5′ to tRNA genes, presumably by suppressing R-loop formation, with integration also dependent on intact debranching activity (see model in Fig. S7).

### Loss of RNase H1 activity increases R-loop formation in mitochondrial DNA

**Figure 4:**
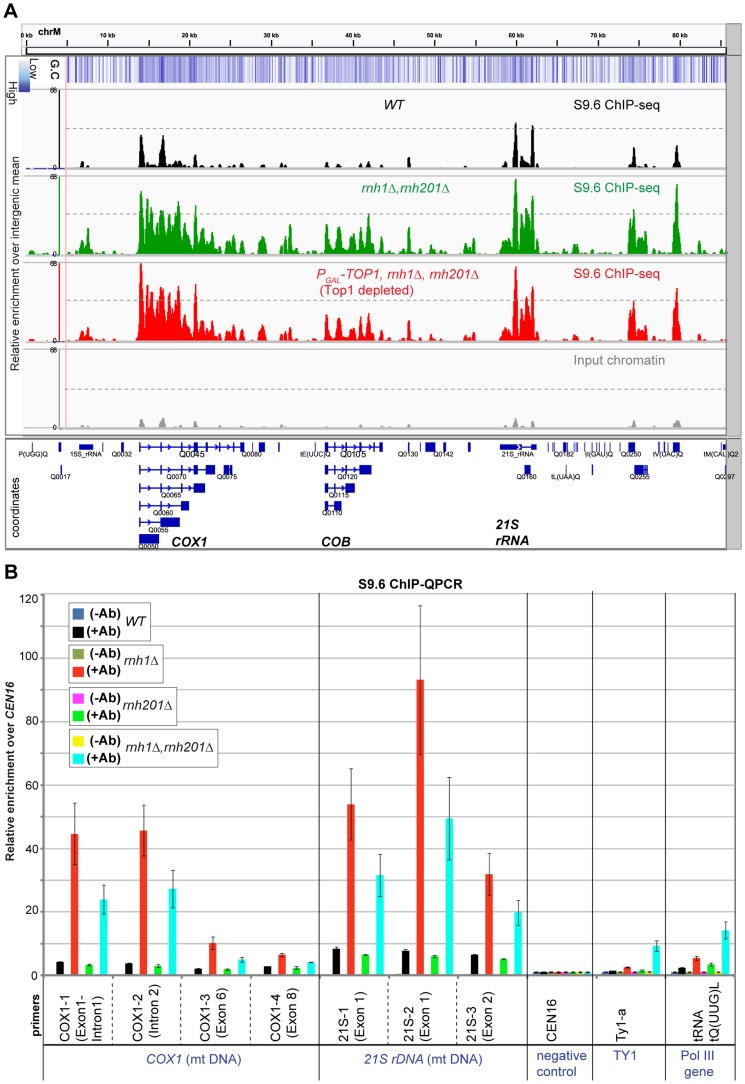
RNase H1 suppresses accumulation of R-loops at mitochondrial DNA. **A**: Profiles of RNA-DNA hybrids over the mitochondrial chromosome (Chr M) in the *WT* (BY4741), the double mutant *rnh1*Δ *rnh201*Δ, and the triple mutant *P_GAL_-TOP1 rnh1*Δ *rnh201*Δ depleted of Top1 for 6 h at 30°C. Input chromatin is shown for the *WT*. A schematic of the mitochondrial transcription units is presented underneath the profiles with the direction of transcription indicated by arrowheads. *COX1* gene ( = *Q0045*) is comprised of 8 exons and 7 introns, *COB* gene ( = *Q0105*) of 6 exons and 5 introns, and *21S rDNA* of 2 exons and 1 intron. Exon and intron sequences are represented by filled boxes and horizontal lines, respectively. The y-axis represents the relative enrichment of reads where values>1 are above the background level of sequencing (i.e. general intergenic mean, see Materials and Methods). G+C content of the DNA sequence was calculated for 100 bp windows and is depicted as a blue intensity. Profiles were generated using the Integrative Genomics Viewer [100]. **B**: ChIP-QPCR analysis of R-loops over mitochondrial genes *COX1* and *21S* rDNA in WT (BY4741) and isogenic single *rnh1Δ*, single *rnh201Δ* and double *rnh1*Δ *rnh201*Δ mutants, grown at 30°C in YEPD medium (glucose 2%). Ty1 retrotransposons and tRNA *tQ(UUG)L* are also shown. Q-PCR values were calculated and normalized to *CEN16* as in Fig. 1A. The mean of three independent experiments is shown with standard error. Ab = antibody S9.6.

Analysis of the S9.6 ChIP-Seq data showed that, unexpectedly, mitochondrial DNA (mtDNA) sequences were enriched with RNA-DNA hybrids (Fig. 4A). At all sites in the mtDNA, R-loop formation was stronger in strains lacking RNase H activity than in the wild-type strain, but this was not further increased by the additional loss of Top1 activity (Figs. 4A and S8). Notably, the degree of R-loop accumulation varied within and between mt transcription units (Figs. 4 and S8, e.g. compare the different regions in the relatively long ∼12.88 Kb *COX1/Q0045* gene with other mt genes in Fig. 4A), possibly reflecting variations in the transcription initiation and elongation rates of mtRNAP [45], as well as differences in the thermodynamic stability of the RNA-DNA hybrids [12], [13]. Reverse transcriptase activity has been reported in mitochondria of *S. cerevisiae* [46]. However, our data show that RNA-DNA hybrids accumulated on mtDNA transcription units are generated through transcription by mtRNAP rather than reverse transcription (Fig. S4).

In order to assess the contributions of RNase H1 and RNase H2 in resolving R-loops in mitochondria, we performed S9.6 ChIP-QPCRs in single *rnh1Δ*, single *rnh201Δ* and double *rnh1*Δ *rnh201*Δ mutants (Fig. 4B). Loss of RNase H1 (+Ab red bars), but not of RNase H201 (+Ab green fluorescent bars), highly increased R-loop levels over the *COX1* and 21S rDNA mt genes. Loss of both RNase H1 and RNase H201 in double mutants resulted in similar or lower levels of R-loop formation than the RNase H1 single mutant (+Ab blue fluorescent bars). Notably, the no antibody controls (−Ab bars) showed no enrichment above background in any strain tested. The recovery of R-loops that mapped to the mtDNA was substantially reduced by treatment of ChIP samples from the WT and double *rnh1*Δ *rnh201*Δ mutant strains with recombinant RNase HI *in vitro* (Fig. 1B), strongly indicating that the mtDNA regions recovered with antibody S9.6 represent *bona fide* sites of R-loop formation.

We conclude that nuclear-encoded RNase H1, but not RNase H2, can degrade R-loops in yeast mitochondria.

### R-loops are enriched at specific sites on intron-containing genes

**Figure 5:**
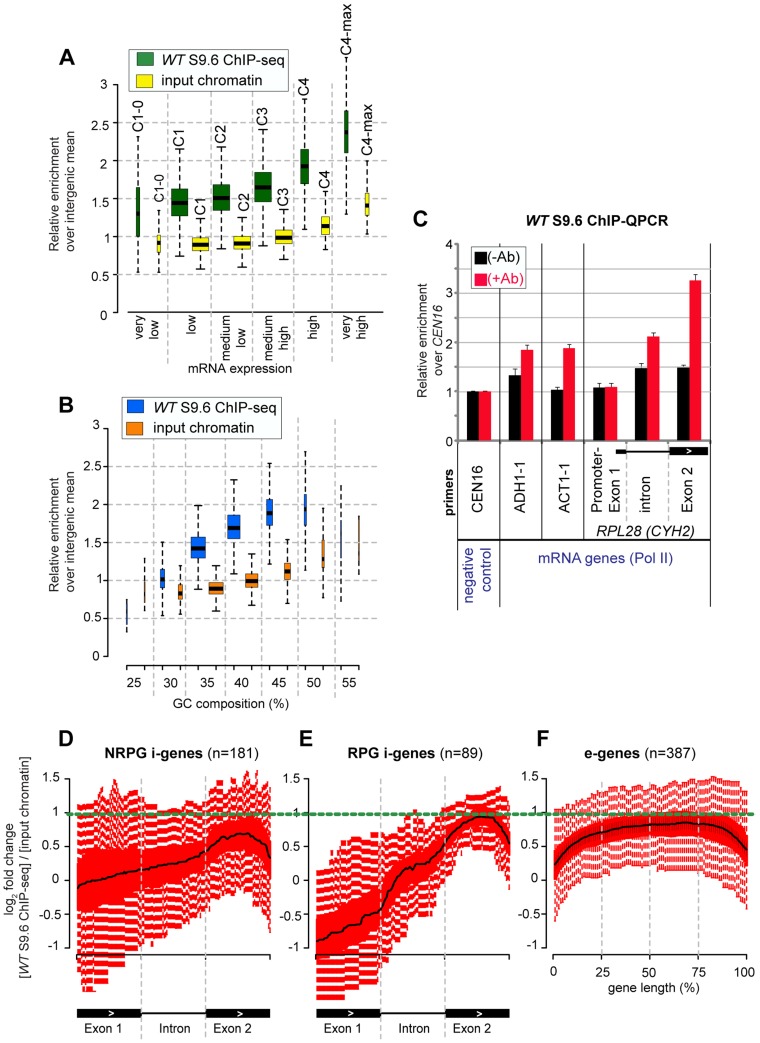
R-loops are enriched over the second exon of intron-containing genes. **A–B**: Box plots of mean sequence read distribution per gene across all yeast protein-coding genes (n = 5864, see Fig. S10A) in samples “input chromatin” and “S9.6 ChIP-seq” from the wild-type strain (BY4741) grown at 30°C in YEPD medium (glucose 2%). The y-axis represents the relative enrichment of sequencing reads where values >1 are above the background level of sequencing (i.e. general intergenic mean, see Materials and Methods). (A) Genes were clustered arbitrarily in 6 main categories based on the strength of their mRNA expression (X-axis): C1-0 (very low), C1 (low), C2 (medium-low), C3 (medium-high), C4 (high) and C4-max (very high) (see Fig. S10A and [101]). We used a Kolmogorov-Smirnov test to determine whether levels of mean sequence distribution differ significantly between the input chromatin and ChIP-seq data within each mRNA expression group: C1-0 (D = 0.5, p-value = 1.85E-008), C1 (D = 0.8109685, p-value = 0), C2 (D = 0.8624161, p-value = 0), C3 (D = 0.8607383, p-value = 0), C4 (D = 0.8439024, p-value = 0) and C4-max (D = 0.7888889, p-value = 4.440892E-16). (B) Genes were clustered based on their GC composition across the entire length of the gene (X-axis). Box-plots show median values (black line) +/−25% quartiles in the box and minimum/maximum distribution of the values (excluding outliers) in the whiskers. The width of the boxes reflects the number of genes in each group. **C**: ChIP-QPCR analysis of R-loops over control *CEN16* and mRNA genes *ADH1*, *ACT1* and intron-gene *RPL28* (*CYH2*) in strain wild-type (BY4741), grown at 30°C in YEPD medium (glucose 2%). Q-PCR values were calculated and normalized to *CEN16* as in Fig. 1A. The mean of three independent experiments is shown with standard error. Ab = antibody S9.6. Exon1 and intron regions of *RPL28* are represented with a filled box and a horizontal line, respectively. **D–F**: Box plots of S9.6 ChIP-seq profiles of R-loops over mRNA genes in the wild-type (BY4741) (same samples as in panel A). (D) Intron-containing, non-ribosomal protein genes (NRPG i-genes). (E) Intron-containing, ribosomal protein genes (RPG i-genes). (F) Top 387 highly expressed intronless genes (e-genes). Each box plot represents the log_2_ fold-change of S9.6 ChIP-seq relative to input chromatin, so the regions above zero value on the Y-axis are enriched with R-loops (see also Fig. S13). For ease of comparison between panels the horizontal dotted green line points to the position of the top R-loop signal on exon 2 in panel E. For i-genes in panels D-E, R-loop profiles are plotted across the Exon1-Intron-Exon2 region. The 5′ end of Exon 1 is defined either as the AUG start codon, or 100 bp upstream of the 5′ splice site for genes with Exon 1 <100 pb (see also Protocol S1). For e-genes in panel F (top 387 highly expressed, see Fig. S10A), R-loop profiles are plotted across the entire length of the gene.

In the wild-type strain, clear enrichment for R-loops in the S9.6 ChIP-seq data relative to the input chromatin was seen at highly expressed mRNA genes (Fig. 5A). Most mRNA genes showing clear enrichment for R-loops also have relatively high G.C contents (Fig. 5B). The ChIP-seq findings were confirmed by ChIP-QPCR for the highly expressed genes *ADH1, ACT1* and *RPL28*, which showed a small but significant enrichment in R-loops (+Ab red bars) relative to no-antibody control (−Ab black bars) (Fig. 5C; see also gene *PMA1* in Fig. S9).

The levels of R-loops detected by ChIP-QPCR at Pol II genes *ACT1*, *ADH1*, *PMA1* and *RPL28* were similar in the wild-type and the double mutant *rnh1*Δ *rnh201*Δ (Fig. S9). Since the numbers of mapped sequences in the S9.6 ChIP-seq samples are expressed relative to total reads, the high increase in signal over Pol III genes, retrotransposons and mitochondria in mutants double *rnh1*Δ *rnh201*Δ and triple *P_GAL_-TOP1 rnh1*Δ *rnh201*Δ is expected to overshadow the effects seen elsewhere, precluding genome-wide analysis of mRNA genes in these strains.

Treatment of S9.6 ChIPs with recombinant RNase HI *in vitro* lead to slight decreases in R-loop signals over all mRNA genes tested (Fig. S9). Note, however, that this is a ChIP experiment in which the RNA, DNA and chromatin are all formaldehyde crosslinked, and these conditions, together with the bound antibody, potentially hinder cleavage of RNA-DNA hybrids by recombinant *E.coli* RNase HI, which has different hybrid hydrolysis properties from eukaryotic RNase H enzymes (for a review on the mechanisms of action of RNase H enzymes see [16]). In the cases of Pol I, Pol III and mtDNA genes (Fig. 1B), RNA-DNA hybrids may be much more accessible to recombinant RNase HI due to the relative lack of nucleosomes at these loci [29], [47], [48],[49] and/or to the length/complexity of the RNA-DNA hybrids [50].

At intron-containing genes (designated here “i-genes”) the distribution of S9.6 ChIP-seq reads was distinctly different over exon and intron sequences (Figs. 5D–E). The density of R-loops was notably higher over the second exon (exon 2) than the first exon (exon 1), or intron, particularly for spliced ribosomal protein genes (RPG i-genes) (Figs. 5D–E). This was confirmed by ChIP-QPCR for the RPG i-gene *RPL28* (Fig. 5C). The majority of yeast i-genes harbor a relatively short exon1 (<150 bp; see Fig. S10B). The asymmetric distribution pattern of R-loops over i-genes, with low relative hit densities over exon 1, was most marked for this class and particularly for the highly expressed RPGs (Fig. S11). This pattern was also visible when individual, well-expressed i-genes were examined (i-genes colored in red in Fig. S12). The distribution of hits along i-genes was very different from that seen on intronless genes (designated here “e-genes”), clearly showing it to be splicing-specific (compare Figs. 5D–E and 5F). Moreover the signal on exon 2 of RPG i-genes was clearly higher than on the RPG e-genes (Fig. S13).

Remapping sequence reads across splice junctions using STAR, revealed no sequenced reads that map across annotated splice junctions in neither the S9.6 CHIP-seq nor the input chromatin. This confirms that R-loops were accumulated over genomic loci and not associated with spliced mRNAs. This is in contrast to RNA-DNA hybrids accumulated over TY1 elements in the double mutant *rnh1*Δ *rnh201*Δ which could be associated with TY1 cDNAs i.e. products of reverse transcription (see Figs. S3, S4).

Analysis of G.C content across i-genes revealed higher enrichment over exon 2 than exon1 and intron regions, in particular the RPGs (Fig. S14). Prediction of the thermodynamic stability patterns of (pre-mRNA)/DNA and DNA/DNA duplexes across i-genes, indicated that (pre-mRNA)/DNA stability is likely to be particularly weak in the intron region adjacent to the 3′ splice site, relative to exon 2, in particular for the RPGs (Fig. S14, for a description of ΔG_9_ calculations see Protocol S1 and [12])). This potentially contributes to the sharp rise in R-loops seen at the intron-exon2 boundary (Figs. 5D–E and S11). Altogether these data clearly show that there is a correspondence between G.C content, thermodynamic stability of (pre-mRNA)/DNA duplexes, R-loop accumulation and transcription activity over yeast i-genes.

On spliced genes, R-loops are less abundant over exon1 and intron sequences than on the second exon, particularly on highly expressed intron-genes. We speculate that R-loops are suppressed at intron regions to ensure proper recognition of 5′ and 3′ splice sites by the splicing machinery, whereas they are favored over exon 2 to decelerate elongation of Pol II and by doing so to promote co-transcriptional splicing [51], [52], [53].

## Discussion

### New RNA-DNA substrates for RNase H

Here we report the application of ChIP-seq to systematically identify sites of RNA-DNA duplex formation throughout the nuclear and mitochondrial genomes in budding yeast. Numerous, prominent sites of R-loop enrichment were identified in actively transcribed genes by all RNA polymerases, Pol I, II, III and mtRNAP. Over the Pol I transcribed rDNA, the distribution of R-loops agreed well with the pattern previously observed in conventional ChIP analyses [23]. In addition, loss of cellular RNase H activity resulted in strong accumulation of RNA-DNA hybrids at Pol III transcribed genes, Ty1 retrotransposons, the second exon of spliced genes and over transcription units of the mtDNA. While this work was in preparation, an analysis of R-loop prone-sites in budding yeast arrived at similar, but not identical, conclusions [11].

### Stable R-loops affect pre-tRNA synthesis

R-loops were strongly detected over the Pol I transcribed 35S rDNA and the 5S rRNA genes in the wild-type. Various other Pol III transcripts including tRNAs, scR1, U6 snRNA and the snoRNA snRNA52 were also enriched in R-loops particularly in the absence of RNase H activities.

Over the Pol I promoter and the rDNA 5′-ETS, R-loop formation was increased in mutants lacking both Top1 and RNase H (data herein and [23]), presumably also reflecting the effects of DNA strand separation over these regions (see Fig. 4 in [25]). The unusual high rates of transcription initiation by Pol I and Pol III (reviewed in [31], [32], [35], [54]) may be facilitated by negative DNA supercoiling and strand separation over the promoter regions [33], [55], [56]. However, this can also favor formation of R-loops that interfere with transcription elongation [23], [25]. The accumulation of negative supercoils behind transcription bubbles is expected to be enhanced by loss of Top1 or also Top2 [23], [25], [33], [57]. Consistent with this, increased pre-tRNA accumulation was observed in strains lacking Top1 or also Top2. However, the increase in pre-tRNAs was reversed when RNase H was also absent. We therefore propose that increased transcription initiation of tRNA genes due to promoter opening, particularly when Top1 (or also Top2) is absent, can be offset by impaired elongation due to stable R-loop accumulation in strains also lacking RNase H. This is reminiscent with the reduced rates of pre-rRNA synthesis in strains lacking both Top1 and RNase H due to stable R-loop accumulation at the rDNA repeats (data herein and [23], [25]).

Our data agree with the recent report [11] that R-loops at tRNA genes are processed by RNase H. It is possible that nascent tRNAs engaged in R-loops in wild-type yeast are rapidly cleaved by RNase H and/or resolved by helicase Sen1/Senataxin [11] and targeted to degradation by the TRAMP/exosome 3′-5′ surveillance machinery [15], [23], [58], [59], [60].

### Roles of Top1 and cellular RNase H in TY1 retrotransposition

Analyses of the Ty1 class of endogenous LTR-retrotransposons in strains lacking cellular RNase H and Top1 activities revealed marked increases in the abundance of RNA-DNA hybrids and in the frequency of retrotransposition.

Most of the RNA-DNA hybrids mapped to TY1 elements in strains lacking cellular RNase H or also Top1 activities are associated with TY1 cDNAs rather than chromosomal genes. These hybrids are products of reverse transcription that may have escaped cleavage by the RT/RNase H protein (for a review on Ty1 replication see [61]). TY1 RNA:cDNA hybrid molecules could be produced during synthesis of either the first (minus) DNA or the second (plus) DNA strand in the VLPs. Minus strand synthesis requires reverse transcription of the highly structured Ty1 genomic RNA, which could be hampered by potential RT pausing/stalling events [62]. Plus strand synthesis requires priming at specific polypurine tracts, which are resistant to cleavage by the RNase H domain of the RT [61].

Ty1 cDNAs and/or retromobility are increased in a variety of different genome-maintenance mutants (reviewed in [36]). It is possible that DNA damage inflicted on the genome by co-transcriptional R-loops, e.g. in mutants lacking cellular RNase H or also Top1, leads to the alleviation of Ty1 dormancy (see model in Fig. 3E). Additionally, RNase H1 and H2 may directly cleave RNA-DNA hybrids generated by reverse transcription of TY1 genomic RNAs. Cleavage of TY1 RNA:cDNA hybrid molecules by cellular RNase H could occur inside and/or outside the VLPs (see model in Fig. 3E and [36], [63]). Notably, RNA-DNA hybrids associated with cDNAs of endogenous retroelements may play roles in the pathogenesis of autoimmune diseases in humans, e.g. in RNase H2-AGS [64], [65].

The LTR-retrotransposons TY3 and TY5 in *S. cerevisiae* and Tf1 in *S. pombe* are selectively targeted to nuclear genomic regions through interactions between the retrotransposon and specific transcription factors and/or chromatin (reviewed in [36], [37], [66]). Ty1 incorporation most commonly occurs by integrase-mediated integration at the nucleosomal H2A/H2B interface upstream of Pol III-transcribed genes [43], [44], [67], with a periodicity of ∼80 bp that is mediated by interactions between the ATP-dependent chromatin remodeling factor Isw2 and the TFIIIB transcription complex [68], [69]. While pre-tRNA synthesis was reduced in strains lacking both Top1 and cellular RNase H activities, the integration of Ty1 at target sites upstream of tRNA^GLY^ was on the contrary strongly increased in these mutants (see model in Fig. 3E). tRNA genes act as nucleosome phasing signals in both directions, possibly due to specific properties of the TFIIIB-TFIIIC transcription complex [49], and R-loops might affect the stability/flexibility of this complex leading to altered nucleosome dynamics/phasing, thus creating an environment that is conducive to Ty1 integration (see model in Fig. S7). It is possible that collisions between the DNA replication machinery and Pol III associated R-loops may also play a role in TY1 integration [70].

### R-loops associate with yeast mitochondrial DNA

We observed the accumulation of R-loops over the mitochondrial DNA (mtDNA) transcription units, specifically in strains lacking RNase H1. The ∼80 Kb *S. cerevisiae* mt chromosome comprises relatively long transcription units such as the genes *COX1/Q0045* and *COB/Q0105*, which expression and polycystronic structure (with multiple exons and introns) are extremely complex [71]. Mitochondrial transcription-translation coupling is expected to suppress R-loop formation, as is believed to be the case in bacteria (reviewed in [72], [73]). However, gene structure and expression, poor packaging of nascent transcripts, G.C composition of the sequence (see Fig. 4A), transcription-mediated topological stress and other factors may favor R-loop formation on mtDNA [4].

The yeast mt DNA encodes proteins and RNAs with key roles in mitochondrial function [74], [75], [76], but most proteins that are needed in mitochondria are encoded by the nuclear genome and imported from the cell cytoplasm. A mitochondrial function for RNase H1 has previously been reported in mammals [19], [77], but this was not known to be the case for yeast. However, dramatic accumulation of R-loops was seen over transcription units in the mtDNA in the absence of RNase H1, but not RNase H2, strongly indicating that RNase H1 does function in this organelle. Notably, the absence of Top1 failed to exacerbate the accumulation of R-loops at mtDNA in yeast strains lacking also RNase H activity.

In yeast the mtDNA is not essential for viability on glucose-containing medium and many mutations cause instability of the mtDNA, leading to a complete loss (rho(0) petites) or truncations (rho(-) petites) of this genome [47], [74]. Yeast strain W303 grows slowly on glycerol-containing medium, on which mtDNA function is required, and shows a relatively high rate (∼15%) of formation of rho(-) petites containing non-functional mtDNA on glucose medium. Loss of RNase H1 in this yeast background increased rho(-) petite formation 2–3 fold, to ∼45% (Fig. S15). Processing of R-loops by RNase H1 may therefore be important for the maintenance and expression of yeast mtDNA.

RNA-DNA hybrids are extensively formed in the circular 16.5 Kb mt chromosome of mammalian cells where they are believed to play important roles during DNA replication [50], [78], and RNase H1 may generate/remove RNA primers during this process [19], [79]. However, the mechanism of mtDNA replication in *S. cerevisiae* is expected to be more similar to the fungus *C. albicans*, which is mediated mainly by recombination-driven replication [80]. The role of RNase H1 in yeast mitochondria may predominately involve the resolution of cotranscriptional R-loops, as in the nuclear genome, rather than direct involvement in the mtDNA replication process.

### Exon 2 is a favored site for R-loop formation in spliced, protein-coding genes

As for RNA Pol I, Pol III and mtRNAP genes, the actively transcribed mRNA genes were also associated with R-loops, albeit at much lower rates. Thus there appear to be a general link between transcriptional activity and R-loop formation in budding yeast, as recently reported in [11].

Competition between RNA packaging and R-loop formation is a normal feature of mRNA synthesis. R-loop formation at mRNA genes could be dictated by factors including increased residence in proximity to the DNA template of poorly packaged transcripts and the G.C content of the sequence (reviewed in [2], [3], [5]). Indeed the thermodynamic helical stability of (pre-mRNA)/DNA duplexes at highly expressed mRNA genes in yeast is higher than DNA/DNA duplexes mostly due to the relatively higher content in G.C of these genes in comparison to the less-well expressed mRNA genes (Fig. S16). More generally, there was a correspondence between transcription activity, R-loop formation and G.C. content at most yeast mRNA genes (see Figs. 5A–B and [11]). Yeast mutants of RNA biogenesis factors, including the helicase Sen1/SENATAXIN, the THO/TREX RNA packaging complexes, the RNA exosome, the RNA-binding protein Npl3 and components of mRNA 3′ cleavage and polyadenylation (mCP) machinery [60], [81], [82], [83], [84], have all been associated with R-loop formation, with deleterious effects on genome stability. In mammalian cells, R-loops can result in silencing of protein coding genes, with potentially pathogenic outcomes [8], [85], [86].

Most intron-containing genes in yeast, particularly ribosomal protein genes, have a short exon 1, and this correlates with higher levels of expression relative to genes with longer exon 1 (see Fig. S10). The close proximity of the 5′ splice site to the promoter region may stimulate transcription via coupling of the splicing and Pol II initiation machineries [87], [88]. In the S9.6 ChIP-seq data, R-loops were reduced over short exon 1 regions and the accompanying intron, relative to the second exon, on most spliced genes (see Figs. 5D–E and S11). It was recently proposed that R-loop formation downstream from CpG-rich regions of strong promoters of highly expressed spliced genes in mammalian cells may be more favored over longer than shorter first exon regions [9]. R-loops can slow down elongation of the RNA polymerase (reviewed in [2], [3]), and co-transcriptional splicing is kinetically coupled to transcription elongation by Pol II (reviewed in [89]). We speculate that co-transcriptional R-loops have been counter selected over short exon 1 and the associated introns. This may promote high expression of these genes, together with proper recognition of their 5′ and 3′ splice sites (SS). Notably, depletion of the splicing factor ASF/SF2 (alternative splicing factor/splicing factor 2) in mammalian cells can lead to increased R-loop formation and genome instability [53], [90]. Whereas R-loops were reduced over exon 1 and intron regions of spliced genes, they were increased over exon 2 (see Figs. 5D–E and S11). We speculate that R-loops over exon 2 could decelerate elongation of Pol II [2], [3], and/or create a chromatin environment favorable for Pol II pausing [89], thus promoting co-transcriptional splicing [51], [52]. Supporting these models, a computational, thermodynamic study covering many genomes including *H.sapiens*, predicted that R-loops will generally be less stable around the 5′ and 3′ SS, due to differences in the helical stability of (pre-mRNA)/DNA and DNA/DNA duplexes [12]. The application of this approach to yeast indicated that (pre-mRNA)/DNA duplexes are indeed intrinsically less favored on introns compared to exons, in particular around the 5′ and 3′ SS (see Fig. S14). Most strikingly, predicted (pre-mRNA)/DNA duplexes are particularly disfavored around the 3′ SS regions of ribosomal protein genes (see Fig. S14), and this is potentially related to the high splicing efficiency of these pre-mRNAs [91].

## Materials and Methods

### Strains, plasmids and growth conditions

Yeast strains and plasmids used in this study are listed in Table S1. Growth and handling of *S.cerevisiae* were by standard techniques. For Top1 and Top2 depletion, cells (*P_GAL_-TOP1* and *P_GAL_-TOP1/2* strains) were grown at 30°C to OD_600_ ∼0.3–0.4 in complete Kaiser synthetic SGS minimal medium (2% galactose, 2% saccharose) then transferred to the pre-warmed complete SD minimal medium (2% glucose). Growth was continued for several hours and maintained in exponential phase by dilution with pre-warmed SD medium.

### Chromatin immunoprecipitation (ChIP) analyses of RNA-DNA hybrids

Immunoglobulins IgG2a of monoclonal antibody S9.6 [26], [27] were purified from mouse hybridoma cell line supernatants by Eurogentec. ChIP of RNA-DNA hybrids using the antibody S9.6 was performed mainly as described in [23]. Crosslinking of exponentially growing cells (OD_600_ ∼0.6, 50 OD_600_/ChIP sample) with formaldehyde (1%) was for 25 min at room temperature. Pellets were resuspended with 400 µL of FA-1 lysis buffer [50 mM HEPES-KOH at pH 7.5, 140 mM NaCl, 1 mM EDTA at pH 8, 1% Triton X-100, 0.1% w/v sodium deoxycholate, plus CPI-EDTA 1× (Protease inhibitor cocktail, Roche 11697498001)], mixed with 500 µL of glass beads (Sigma, G8772), and vortexed (Vortex Genie 2T, Scientific Industries) for 45 min at full speed at 4°C. Glass beads were removed and cross-linked chromatin was recovered by centrifugation at full speed for 10 min at 4°C (supernatant discarded). Eight-hundred microliters of FA-1 buffer were added on the top of the pellet. Sonication of chromatin was performed for 2 min (10 sec ON, 15 sec OFF, 20% amplitude; Branson Digital Sonifier) to yield an average DNA fragment size of ∼500 bp. Sonicated chromatin were spun for 15 min at full speed at 4°C and glycerol 5% was added to supernatants. Sonicated chromatin were mixed with sepharose Cl-4B beads (Sigma CL4B200) and cleared for 1 h at 4°C. Twenty microliters were kept for control Input chromatin. Immunoprecipitations were performed by mixing ‘cleared-sonicated chromatin’ with 35–40 µg of IgG2a of antibody S9.6 together with 100 µl bed of Protein A sepharose CL-4B beads (GE Healthcare 17-0780-01) on a rotating wheel overnight at 4°C. To assess contribution of background, a ‘beads-only’ internal control was prepared in parallel to immunoprecipitated samples but without addition of any antibody. Beads were recovered (see also paragraph ‘Treatment of S9.6 ChIP with recombinant RNase H’) and washed successively with FA-1 buffer (plus CPI-EDTA 1×), FA-2 buffer (as FA-1 buffer but with 500 mM NaCl, plus CPI-EDTA 1×), FA-3 buffer (10 mM Tris-HCl at pH 8, 0.25 M LiCl, 0.5% NP-40, 0.5% w/v sodium deoxycholate, 1 mM EDTA at pH 8, plus CPI-EDTA 1×), and TE 1× (100 mM Tris-Cl at pH 8, 10 mM EDTA at pH 8) at 4°C. Cross-link reversal of sonicated- chromatin from samples ‘input chromatin’, ‘beads-only’ and ‘S9.6 immunoprecipitate’ were performed by incubating the washed beads overnight at 65°C in 250 µL of TE buffer containing 1% SDS and 1 mg/mL proteinase K. DNA was purified using Qiagen PCR purification kit and eluted with 55 µL of buffer EB containing RNase A (0.5 µg/mL). 10–20 ng/µl DNA were recovered from ‘S9.6 immunoprecipitates’ from wild-type cells as measured by Qubit dsDNA HS Assay Kit (Invitrogen, Q32851). Quantitative PCRs (qPCRs) were performed in triplicate in a MX3005P real-time PCR machine (Agilent Technologies) in 10 µl reaction containing: 5 µl of 2× TaKara SYBR premix Ex Taq II Tli Rnase H Plus (Clontech RR820L), 1 µl DNA (but 10% of input chromatin), 0.4 µl of 10 µM primers (see Table S3), 0.04 µl of Rox II and 3.56 µl of water. Values for ChIPs were calculated using the formulas ΔΔCt “no antibody” = 2^−(Ct ‘beads only’ - Ct ‘input chromatin’)^ and ΔΔCt “S9.6 immunoprecipitate” = 2^−(Ct ‘S9.6 immunoprecipitate’ - Ct ‘input chromatin’)^. The ‘S9.6 immunoprecipitate’ and ‘input chromatin’ were further processed for ChIP-seq as described below.

### Preparation of Sollexa/Illumina libraries for S9.6 ChIP-seq

200 ng of DNA (‘S9.6 immunoprecipitate’ and ‘input chromatin’) were linker-ligated as described mainly in the Illumina's hand book with some modifications. Step1 “Repair DNA ends”: 100 µl reactions contained, DNA, 1× T4 DNA ligase buffer (NEB, B0202S), 0.4 mM dNTP mix, 15 units T4 DNA polymerase (NEB, M0203L), 50 units T4 Polynucleotide Kinase (NEB, M0201L) and 5 units DNA Polymerase I- Large (Klenow) Fragment (NEB, M0210). Reactions were incubated at 20°C for 30 min. DNA was purified using QIAquick PCR Purification Kit (Qiagen, 28106). DNA columns were centrifuged several times up to 10 min to ensure that no residual traces of ethanol are left in the column. DNA was eluted with 32 µl EB buffer (10 mM Tris-HCl, pH 8.5) which was pre-heated to 55°C. Step2 “Add A”: 50 µl reactions contained DNA from step 1, 1× NEB buffer 2 (NEB, B7002S), 0.2 mM dATP and 15 units Klenow Fragment 3′→5′ exo minus (NEB, M0212L). Reactions were incubated for 30 min at 37°C. DNA was purified using MinElute spin column (Qiagen, 28006) and eluted with 10 µl EB buffer as described in step 1. Step 3 “Ligation with standard pair-end (PE) adapters”: 30 µl reactions contained DNA from step 2, 1× Quick DNA ligase buffer (NEB, M2200S), 3.33 nM PE adapter mix and 1600 units Quick T4 DNA ligase (NEB, M2200S) (for primer sequences see Table S3). Reactions were incubated at room temperature (18–22°C) for 30 min. DNA was purified and eluted with 36.5 µl EB buffer as described in step 1. Step 4 ‘PCR amplification’: 50 µl reactions contained: DNA from step three, 1 unit Phusion high fidelity (HF) DNA polymerase (NEB, B05185), 1× HF buffer (NEB, B05185), 0.2 µM ‘primer 1.2’, 0.2 µM ‘primer 2.2’ and 0.2 mM dNTP mix (for primer sequences see Table S3). Cycling conditions were 98°C for 30 sec; followed by 18 cycles (98°C for 10 sec, 65°C for 30 sec and 72°C for 1 min); followed by 72°C for 5 min; followed by cooling to 4°C. PCR DNA was purified using MinElute spin column (Qiagen, 28006) and eluted with 10 µl EB buffer as described in step 1. Step 5 “size selection”: DNA from step 4 was well resolved on 2% agarose gel [mixture 3::1 of standard agarose:: Metaphor agarose (Lonza, 50180)] in 1× TBE, alongside with a DNA ladder, stained with SYBR safe (Invitrogen) and visualised with Fuji FLA-5100 PhosphorImager. 300±50 bp DNA were excised from the gel and purified using QIAquick Gel Extraction Kit (Qiagen, 28706). Note that in order to improve the representation of A+T rich-DNA sequences agarose gel slices were melted at room temperature (18–22°C). DNA was eluted using MinElute spin column (Qiagen, 28006) with 10 µl EB buffer as described in step 1 and sent for high-throughput sequencing.

### Treatment of S9.6 ChIP with recombinant RNase H

One-hundred microliters of bed of Protein A beads incubated with sonicated-crosslinked-chromatin and antibody S9.6 (see paragraph ‘Chromatin immunoprecipitation analyses of RNA-DNA hybrids’) were washed successively with FA-1 buffer (plus CPI-EDTA 1×), TE 1× buffer (plus CPI-EDTA 1×) and 10 mM Tris-HCl pH 8 (plus CPI-EDTA-free 1×, Roche 11873580001). Washed beads were re-suspended in 300 µl of reaction buffer containing RNAse H buffer 1× (NEB, M0297L), 4% glycerol and 20 µg/ml BSA. Beads were incubated for 2.5 h at 37°C in absence or presence of 15 µl of recombinant *E. coli* RNase HI (75 units, NEB, M0297L), with shaking at 1000 rpm (Eppendorf Thermomixer). RNase H reactions were stopped by adding 10 mM EDTA. Beads were washed successively with buffers FA-2, FA-3, and TE 1×, sonicated-chromatin was reverse-crosslinked, and DNA was recovered and analysed by q-PCR as described for the standard ChIP protocol.

### Northern analyses

Equal amounts of total RNA (10 µg) were resolved on standard 8% polyacrylamide-8.3M urea gels for low molecular weight RNAs. Northern hybridizations for tRNAs were performed overnight at 37°C in ULTRAHyb-oligo buffer (Ambion, Invitrogen, AM8663) and washes done at 37°C in SSC 6×. Northern signals were generated by a Fuji FLA-5100 PhosphorImager and quantified with AIDA software (Raytest). For primer sequences see Table S2.

### Ty1 retromobility assay

In strain JC3212 (BY4741, *TY1his3AI-[Δ1]-3114*) *HIS3* gene was inserted in the *TYB* sequence of a Ty1 element in the antisense orientation (see Table S1 and [41]). *HIS3* RNA synthesis from *TY1his3AI* element was interrupted by an artificial *AI* intron which is only spliced during transcription of *TY1* RNA. *Ty1his3AI* retromobility occurs only when the *Ty1his3AI* RNA is spliced, reverse transcribed and the resulting *Ty1HIS3* cDNA is incorporated into the genome by integration or recombination. For the activation of Ty1 retrotransposition in conditions non-permissive for Top1 expression (Figs. 3C–D), cells were: 1) grown overnight at 30°C in Kaiser synthetic SGS Drop-Out minimal medium, 2) diluted in SD Drop-Out medium to OD_600_ ∼0.05 and grown for 3 doublings at 30°C, 3) re-diluted to OD_600_ ∼0.01 in the same medium and aliquoted in 5 cultures of 10 ml each, and, 4) grown at 18°C until saturation (4–6 d). Cells were harvested, washed and re-suspended in 5 ml sterile water. For total number of colonies, aliquots of each culture (dilution 1∶10^6^) were plated on SD Drop-Out -Leu minimal medium and incubated at 30°C. For HIS+ colonies, aliquots of each culture (dilution 1∶2) were plated on SD Drop-Out -Leu-His plates and incubated at 30°C. The rate of Ty1*his3AI* transposition is the number of HIS+ colonies divided by the total number of colonies (as described in [92]).

### Integration assay for Ty1 elements at tRNA^GLY^ sites

Spontaneous Ty1 insertions upstream of 16 tRNA^GLY^ genes were detected as described previously [93], [94] with some modifications. To confirm that the genomic DNA samples were in the linear range for PCR, DNA concentrations were measured with a Qubit dsDNA BR Assay Kit (Invitrogen, Q32850) and equal amounts of 6 or 30 ng DNA were assayed by PCR. Reactions of 50 µl contained 1× Phusion HF buffer (NEB, B05185), 0.2 µM primer ‘TYB OUT’, 0.2 µM primer ‘SUF16’ (for primer sequences see Table S3), 0.2 mM dNTP mix, 1 unit Phusion HF (NEB, B05185), and genomic DNA. Cycling conditions were 98°C for 30 sec; followed by 30 cycles (98°C for 10 sec, 57°C for 30 sec and 72°C for 1 min); followed by 72°C for 10 min; followed by cooling to 4°C. PCR DNA fragments were resolved on a standard 1.5% agarose gel (stained with SYBR safe, Invitrogen) in 1× TBE and visualized with a Fuji FLA-5100 PhosphorImager. For primer sequences see Table S3.

### Southern blot analysis of Ty1 cDNA

Total genomic DNA were extracted by standard glass-bead/phenol lysis (e.g. see [95]). DNA concentrations were measured with a Qubit dsDNA BR Assay Kit (Invitrogen, Q32850). 2 µg DNA were incubated overnight at 37°C in presence of 200 units of restriction endonuclease PvuII-HF (NEB, R3151). DNA samples were resolved on a standard 1% agarose gel (stained with ethidium bromide) in 1× TBE. Washes and blotting of the gel were performed mainly as described in [95], but the depurination step was omitted. DNA random priming probes were prepared using DECAprime II Random Primed DNA Labelling Kit (Ambion, Invitrogen, AM1455; for primer sequences see Table S2), hybridized overnight at 42°C in Hybridization Buffer (50% formamide, 5× SSC, 5× Denhardt's solution, 0.5% SDS and 100 µg/ml sonicated salmon sperm DNA), and washed at 55°C with 2× SSC, 0.1% SDS and 0.1× SSC, 0.1% SDS. Southern signals were generated by a Fuji FLA-5100 PhosphorImager and quantified with AIDA software (Raytest).

### Western blotting

Total protein extracts and Western blot analysis were performed using standard procedures. Mouse anti-Ty1 Gag antibodies, raised against the Glu-Val-His-Thr-Asn-Gln-Asp-Pro-Leu-Asp peptide (Diagenode, anti-Ty1-tag, MAB-054-050; and see [96]), and rabbit anti-beta-actin antibodies (Abcam, ab 34731) were used as primary antibodies. Horseradish peroxidase-conjugated antibodies (GE Healthcare) were used as secondary antibodies.

### Bioinformatic analysis

#### Read alignment and normalization

50 bp reads were sequenced by an Illumina MiSeq Benchtop Sequencer, quality trimmed and filtered with Trimmomatic and aligned to the *S.cerevisiae* sacCer3 genome assembly using Novoalign V2.07. Reads mapping to more than 1 location were assigned a random alignment (or uniquely mapped for tRNA heatmaps in Figs. 1C–D). Reads aligning to rDNA (except for Fig. S1) were further removed from the analysis. Alignments (BAM files) were transformed into coverage files (bigWig) using the deepTools package, which extends reads to the estimated fragment length (200 bp) and applies a windowing function to calculate the average read depth in 50 bp windows. A smoothing function was also applied so each 50 bp window contained the average value of the surrounding 100 bp. In order to compensate for differences in ChIP-seq efficiencies a background level of sequencing (see e.g. Fig. S12) was attained by calculating the mean read depth of all intergenic regions covered by at least 1 read (regions not annotated by SacCer3 genome http://www.yeastgenome.org). A measure of enrichment for each window was given as the ratio of the windows mean depth vs the intergenic mean (i.e. 50 bp window score = “average read depth in surrounding 100 bp”/“intergenic mean depth”). This method of normalization was mainly adapted from [97].

### Accession numbers

The data discussed in this publication have been deposited in NCBI's Gene Expression Omnibus [98] and are accessible through GEO Series accession number GSE53420.

## Supporting Information

Figure S1 R-loops over the rDNA repeats are detected by S9.6 ChIP-seq. A: Analysis of R-loops by ChIP-seq using antibody S9.6 in the wild-type strain BY4741 (*WT*) and double mutant *rnh1*Δ *rnh201*Δ, and in triple mutant *P_GAL_-TOP1 rnh1*Δ *rnh201*Δ depleted of Top1 for 6 h at 30°C. Also shown is the input chromatin profile of the wild-type strain. Raw read counts were not corrected by the number of rDNA repeats. G+C content of the DNA sequence was calculated for 100 bp windows and is depicted as a blue intensity. Shown below the profiles is a diagram of one rDNA repeat which comprises the 35S rDNA gene, transcribed by Pol I in to the 35S pre-rRNA which is processed to 18S, 5.8S, and 25S rRNAs. 35S genes are flanked by the intergenic spacers IGS1 and IGS2 and the 5S rDNA gene, transcribed by Pol III. The direction of transcription is indicated by a tailed arrow. Chr = chromosome. Prominent R-loop peaks discussed in the text are highlighted by stars. ETS = external transcribed spacer. ITS = internal transcribed spacer. Profiles were generated using Integrative Genomics Viewer [100]. B: Relative recovery of rDNA sequences in the “input chromatin” and “S9.6-immunoprecipitates.”
(TIF)

Figure S2 Average profiles of RNA-DNA hybrids over all Ty1 elements. Median S9.6 ChIP-seq profiles of RNA-DNA hybrids over the 31 Ty1 elements in strains *WT* (BY4741) and double mutant *rnh1*Δ *rnh201*Δ, and in triple mutant *P_GAL_-TOP1 rnh1*Δ *rnh201*Δ depleted of Top1 for 6 h at 30°C. Median profiles of control input chromatin from *WT* are also shown. The y-axis represents the relative enrichment of reads where values >1 are above the background level of sequencing (i.e. general intergenic mean, see Materials and Methods).
(TIF)

Figure S3 TY1 cDNAs accumulate in mutants lacking cellular RNase H or also depleted of Top1. A: Diagrams of unintegrated Ty1 cDNA and a genomic Ty1 element, indicating the location of the *TY1B* hybridization probe (filled black rectangle) and relevant *Pvu*II cleavage sites. Probe *TY1B* detects an ∼2 Kb PvuII DNA fragment of unintegrated Ty1 cDNA and variably sized PvuII DNA fragments >2 Kb containing the junction of Ty1 elements with chromosomal DNA at different locations in the genome. B: Southern analyses of Ty1 cDNA from strain *WT* (BY4741) and double mutant *rnh1*Δ *rnh201*Δ, grown at 22°C in YEPD medium (glucose 2%) until saturation, in the absence or presence of 600 µg/ml of phosphonoformic acid (PFA), which is an inhibitor of TY1 RT [39]. The ratio of the ∼2 Kb Ty1 cDNA was determined by normalising the intensity of the Ty1 cDNA band relative to the average intensities of 3 genomic Ty1 junction bands (filled black circles). Values were expressed relative to the wild-type (-PFA, lane 1) which was set to 1. TY1 cDNA band is indicated by a tailed arrow. Indicated to the left of the gel the migration position of a 2 Kb band from a DNA size ladder. C: Southern analyses of Ty1 cDNAs from strain *WT* (BY4741) and mutant strains double *rnh1*Δ *rnh201*Δ, single *P_GAL_-TOP1*, triple *P_GAL_-TOP1 rnh1*Δ *rnh201*Δ, single *dbr1Δ*, triple *rnh1Δ rnh201Δ dbr1Δ*, double *P_GAL_-TOP1 dbr1Δ*, and quadruple *P_GAL_-TOP1 rnh1Δ rnh201Δ dbr1Δ*. Cultures were grown at 30°C in medium containing galactose and sucrose (permissive for *P_GAL_-TOP1* expression, lanes 1–8) and shifted for 6 h to medium containing glucose (non-permissive for *P_GAL_-TOP1* expression, lanes 9–16). TY1 cDNA band is indicated by a tailed arrow. Indicated to the left of the gel the migration position of a 2 Kb band from a DNA size ladder.
(TIF)

Figure S4 Reduced accumulation of RNA/DNA hybrids at Ty1 in the absence of RT activity. Double mutant *rnh1*Δ *rnh201*Δ was grown in YEPD medium (glucose 2%) at 22°C in the absence or presence of 600 µg/ml of phosphonoformic acid (PFA), which is an inhibitor of TY1 reverse transcriptase (RT) [39]. ChIPs were performed with no-antibody (−Ab) or antibody S9.6 (+Ab). The Pol I transcribed gene (18S rDNA), Ty1 retrotransposons, mtDNA transcription units (*COX1* and 21S rDNA), Pol III gene tRNA *SUF2*, mRNA gene *RPL28* and *CEN16* were analyzed by Q-PCR as described in Fig. 1A. The mean of three independent experiments is shown with standard error.
(TIF)

Figure S5 Gag proteins are slightly increased in mutants lacking both Top1 and cellular RNase H. Immunoblots of cellular homogenates from strain *WT* (BY4741) and mutant strains double *rnh1*Δ *rnh201*Δ, triple *P_GAL_-TOP1 rnh1*Δ *rnh201*Δ and quadruple *P_GAL_-TOP1 rnh1Δ rnh201Δ dbr1Δ*. Yeast cultures grown at 30°C were shifted from galactose- and sucrose- containing-medium to glucose medium and harvested at 6 h depletion of Top1. *Panel I*. Blot probed with antibody anti-Ty1 Gag. The Gag-p49 and processed-Gag-p45 bands each appear as doublets. *Panel II*. Blot probed with antibody anti-beta-actin.
(TIF)

Figure S6 PCR analyses of integration of Ty1 at *tRNA^GLY^* in mutants lacking RNase H and/or Dbr1. Four independent isolates for each strain, *WT* (BY4741) and mutant strains single *dbr1Δ*, double *rnh1*Δ *rnh201*Δ, triple *rnh1Δ rnh201Δ dbr1Δ*, triple *P_GAL_-TOP1 rnh1*Δ *rnh201*Δ, and quadruple *P_GAL_-TOP1 rnh1Δ rnh201Δ dbr1Δ*, were grown until saturation at 18°C in medium containing both galactose and sucrose (permissive for *P_GAL_-TOP1* expression). *Panel I*. See legend in Fig. 3D. *Panel II*. Representative examples of SYBR-stained gels are shown, revealing integration of Ty1 cDNA upstream of the 16 *tRNA^GLY^* gene loci. Shown to the right of the gels DNA ladders with lengths in base-pairs (bp).
(TIF)

Figure S7 Model: Pol III-associated R-loops facilitate targeting of TY1 at 5′ flanking regions of tRNA genes. Ty1 integration upstream of tRNA genes is specifically targeted to the H2A/H2B interface of nucleosomal DNA in a ∼1 kb window [43], [44], [67]. The nascent transcript behind elongating Pol III can invade the DNA duplex and hybridize with the DNA template strand, generating a three-stranded R-loop structure, composed of an RNA-DNA duplex and an unpaired non-template DNA strand. We postulate that alterations in chromatin structure due to R-loop formation [102], [103] at Pol III genes, favor recruitment of the TY1 pre-integration complex formed by the integrase (IN) and the cDNA (green thick arrow = positive regulation). Black thick arrow = transcription direction. The diagram is not drawn to scale.
(TIF)

Figure S8 ChIP-QPCR of R-loops at mtDNA in mutants lacking both Top1 and cellular RNase H. ChIP samples using antibody S9.6 (same as in Fig. 3A) are from strains *WT* (BY4741) double mutant *rnh1*Δ *rnh201*Δ and from mutants triple *P_GAL_-TOP1 rnh1*Δ *rnh201*Δ and quadruple *P_GAL_-TOP1 rnh1Δ rnh201Δ dbr1Δ* depleted of Top1 for 6 h at 30°C. *CEN16* and four different regions of *COX1* gene were analysed by Q-PCR as described in Fig. 1A. Ab = antibody S9.6.
(TIF)

Figure S9 *Formaldehyde*-crosslinked R-loops associated with mRNA genes are slightly cleaved *in vitro* by recombinant RNase HI. ChIP samples are from strains *WT* (BY4741) and double mutant *rnh1*Δ *rnh201*Δ (same as in Fig. 1B) grown at 30°C in YEPD (glucose 2%). ChIPs were performed with no-antibody (−Ab) or antibody S9.6 (+Ab), or with antibody S9.6 but beads were further incubated for 2.5 h at 37°C in absence [(+Ab)/(−RNase H)] or presence [(+Ab)/(+RNase H)] of recombinant RNase HI (see Material and Methods). *CEN16*, the highly Pol II transcribed mRNA genes *ADH1*, *ACT1*, *PMA1* and *RPL28* (exon 2), and the telomeric region *Tel01L*, were analyzed by Q-PCR as described in Fig. 1A.
(TIF)

Figure S10 Expression of spliced genes with a “short-exon1” is generally higher than those with a “long-exon1.” A: Plot of RNA expression of yeast mRNA genes. Panel **I**. Raw transcriptome sequencing (RNA-seq) reads of exponentially growing wild-type strain BY4741 [101] were processed as described in Protocol S1. Normalised reads per base of exon of the 5864 protein-coding genes were plotted on the Y-axis. The top value of the Y-axis was arbitrarily set to 15. We clustered the mRNA genes into four groups of RNA expression, and indicated their boundaries by red vertical lines on the plot: C1 (low, n = 1788), C2 (medium-low, n = 1788), C3 (medium-high, n = 1788) and C4 (high, n = 500). The seventy four very-lowly expressed mRNA genes (subgroup “C1-0” in Fig. 5A) were included in group C1. The top ninety very-highly expressed mRNA genes (subgroup “C4-max” in Fig. 5A) were included in group C4. Ribosomal protein genes (RPG) and non-ribosomal protein genes (NRPG) are represented by a green or black vertical line, respectively. The plot region featuring the 500 highly expressed mRNA genes of group C4 including all the RPGs is magnified in panel **II**. n = number of genes. B: Plot of yeast mRNA intron-genes (i-genes) grouped according to the length of their first exon (exon 1) (panel **I**). The region of the plot featuring the i-genes with Exon 1 <300 bp is magnified in panel **II**: 87 RPGs (out of 89 i-genes) and 144 NRPGs (out of 181 i-genes) have an Exon 1 <300 bp. C: Boxplots of RNA-seq and Net-seq data from wild-type strain BY4741 [101] covering the 181 i-genes of NRPG (the 3 dubious open reading frames *YDR535C*, *YLR202C* and *YOR318C* were excluded from our analysis in Figs. 5, , S11, S13, S14), which were divided in to two groups based on the length of Exon1 (<100 and >100 bp). Normalised average read coverage of RNA-seq and NET-seq data (the number of reads per base of exon; see Protocol S1 and [101]) were calculated for each i-gene and the two Exon1-groups were represented as boxplots. Box-plot representation shows median values (black line) +/−25% quartiles in the box and minimum/maximum distribution of the values (excluding outliers) in the whiskers. We used a Kolmogorov-Smirnov test to show that levels of expression differ significantly between the two groups of Exon 1 (<100 and >100): RNA-seq (D = 0.4426, p-value = 1.392e-06) and NET-seq (D = 0.3463, p-value = 0.0002232). n = number of genes.
(TIF)

Figure S11 R-loop distribution over mRNA spliced genes according to the length of their first exon. Average profiles of S9.6 ChIP-seq and input chromatin of mRNA intron-genes (i-genes) in the wild-type strain (BY4741), grown at 30°C in YEPD medium (glucose 2%). Averaged reads were plotted on sequences encompassing Exon1-intron-Exon2 regions as described in Protocol S1. The 5′ end of Exon 1 is defined either as the AUG start codon, or 100 bp upstream of the 5′ splice site for genes with Exon 1 <100 pb (see also Protocol S1). The i-genes were split in to the ribosomal protein genes (RPG) (panel A) and the non-ribosomal-protein genes (NRPG) (panel B), and further segregated in to four sub-categories according to the length of their first exon (Exon 1): (0–50 bp), (50–100 bp), (100–150 bp) and (>300 bp). The y-axis represents the relative enrichment of reads where values>1 are above the background level of sequencing (i.e. general intergenic mean, see Materials and Methods).
(TIF)

Figure S12 Examples of S9.6 ChIP-seq profiles of mRNA genes. Profiles of input chromatin and S9.6 ChIP-seq are for the wild-type strain (BY4741) grown at 30°C in YEPD medium (glucose 2%). Shown for spliced genes *RPL28* (A), *ACT1* (B) and *EFB1* (E) and intronless genes *SEH1* (A), *FET5* and *YPT1* (B), *DUF1*, *MHF1* and *ADH1* (C), *LEU1* and *PMA1* (D), *SSA1* and *VPS8* (E), and *IBA57*, *RPS5* and *ENT3* (F). The y-axis represents the relative enrichment of reads where values >1 are above the background level of sequencing (i.e. general intergenic mean, see Materials and Methods). G+C content of the DNA sequence was calculated for 100 bp windows and is depicted as a blue intensity. Shown below the profiles is a graphical representation of genomic features, with exon and intron sequences depicted as filled boxes and horizontal lines, respectively. The direction of transcription is indicated by a tailless arrow. Highly expressed genes are colored in red. Chr = chromosome. Profiles were generated using Integrative Genomics Viewer [100].
(TIF)

Figure S13 Comparison of R-loop distribution across Exon 2 of spliced-genes and across non-spliced ribosomal protein genes. Box plots of mean sequence read distribution of R-loops per gene across the second exon of the i-genes NRPG (I) and RPG (II), and the entire length of the e-genes RPG (III). Each box plot represents the log2 fold change of mean S9.6 ChIP-seq relative to input chromatin in the wild-type (BY4741), grown at 30°C in YEPD medium (glucose 2%). Box-plot representation shows median values (black line) +/−25% quartiles in the box and minimum/maximum distribution of the values (excluding outliers) in the whiskers (the regions above zero value on the Y-axis are enriched with R-loops; see also Fig. 5). n = number of genes. RPG = ribosomal protein genes. NRPG = non-ribosomal protein genes. i-genes = intron-containing genes. E-genes = intronless genes. We used a Kolmogorov-Smirnov test to determine whether levels of log2 fold change of mean sequence distribution differ significantly between box plots I and II (D = 0.449314048047675, p-value = 6.90119072999096e-11), and II and III (D = 0.39559925093633, p-value = 6.55907560322966e-05).
(TIF)

Figure S14 Thermodynamic helical stability of DNA/DNA and (pre-mRNA)/DNA duplexes of yeast mRNA intron-genes. The mRNA intron-genes (i-genes) were split in to the non-ribosomal-protein genes (NRPG) (**A**) and the ribosomal protein genes (RPG) (**B**). The thermodynamic helical stability of polynucleotide sequences (ΔG_9_ values of DNA/DNA and (pre-mRNA)/DNA duplexes) and the concentrations of G.C nucleotides were calculated for non-overlapping windows of 9 bases as described in Protocol S1 and [12]. Averaged values of ΔG_9_ and [G+C] nucleotides for the i-genes in each group were plotted on sequences encompassing Exon1-intron-Exon2 regions as described in Protocol S1. The 5′ end of Exon 1 is defined either as the AUG start codon, or 100 bp upstream of the 5′ splice site for genes with Exon 1 <100 pb (see also Protocol S1). n = number of genes. 5′SS = 5′ splice site. 3′SS = 3′ splice site.
(TIF)

Figure S15 Petite frequency is higher in yeast *rnh1Δ* mutants than in isogenic wild-type W303a. Strains wild-type W303a and mutant *rnh1Δ* were grown at 30°C in YEP medium containing 3% glycerol. After four days cells were washed with sterile water, diluted appropriately, plated onto YEP medium containing 2% glucose and incubated at 30°C. One feature of ‘petite’ *ade2^−^* cells is to have a small size and the inability to convert an intermediate in the adenine biosynthesis pathway (AIR) into a red pigment due to impaired respiratory functions (e.g. see [104]). Conversely, ‘grande’ *ade2^−^* cells have a relatively large size and accumulate a red pigment due to functional mitochondria. For each strain a total of 8000 colonies were counted and petite/white and grande/red colonies were scored. Values are means of ten independent isolates with standard errors.
(TIF)

Figure S16 Thermodynamic helical stability of DNA/DNA and (pre-mRNA)/DNA duplexes of yeast mRNA intronless genes. The mRNA intronless genes (e-genes) were split in to four groups according to their mRNA expression: panel A (group C1, low expression, n = 1674), panel B (group C2, medium-low expression, n = 1754), panel C (group C3, medium-high expression, n = 1708) and panel D (group C4, high expression, n = 387). For *Δ*G_9_ values of DNA/DNA and (pre-mRNA)/DNA duplexes and [G+C] nucleotides see Fig. S14 and Protocol S1. Averaged values of *Δ*G_9_ and [G+C] for the e-genes in each group were plotted on sequences encompassing the entire length of the gene (0–100%). n = number of genes.
(TIF)

Table S1 List of strains and plasmids. A: Strains. B: Plasmids.
(DOC)

Table S2 Oligonucleotides used in Northern and Southern analysis. A: Oligonucleotides for Northern analysis of tRNAs and rRNAs. B: Oligonucleotides for Southern analysis of Ty1 cDNAs.
(DOC)

Table S3 Oligonucleotides used in ChIP-QPCR, PCR and ChIP-seq analysis. A: ChIP-QPCR primers. B: Standard PCR primers. C: ChIP-seq primers.
(DOC)

Protocol S1 Bioinformatic analysis.
(DOC)
