# Supplementary figures and images for: Genome-Wide Distribution of RNA-DNA Hybrids Identifies RNase H Targets in tRNA Genes, Retrotransposons and Mitochondria
Source: PLoS Genet. 2014 Oct 30;10(10):e1004716. doi: 10.1371/journal.pgen.1004716 (PMC4214602; doi:10.1371/journal.pgen.1004716)

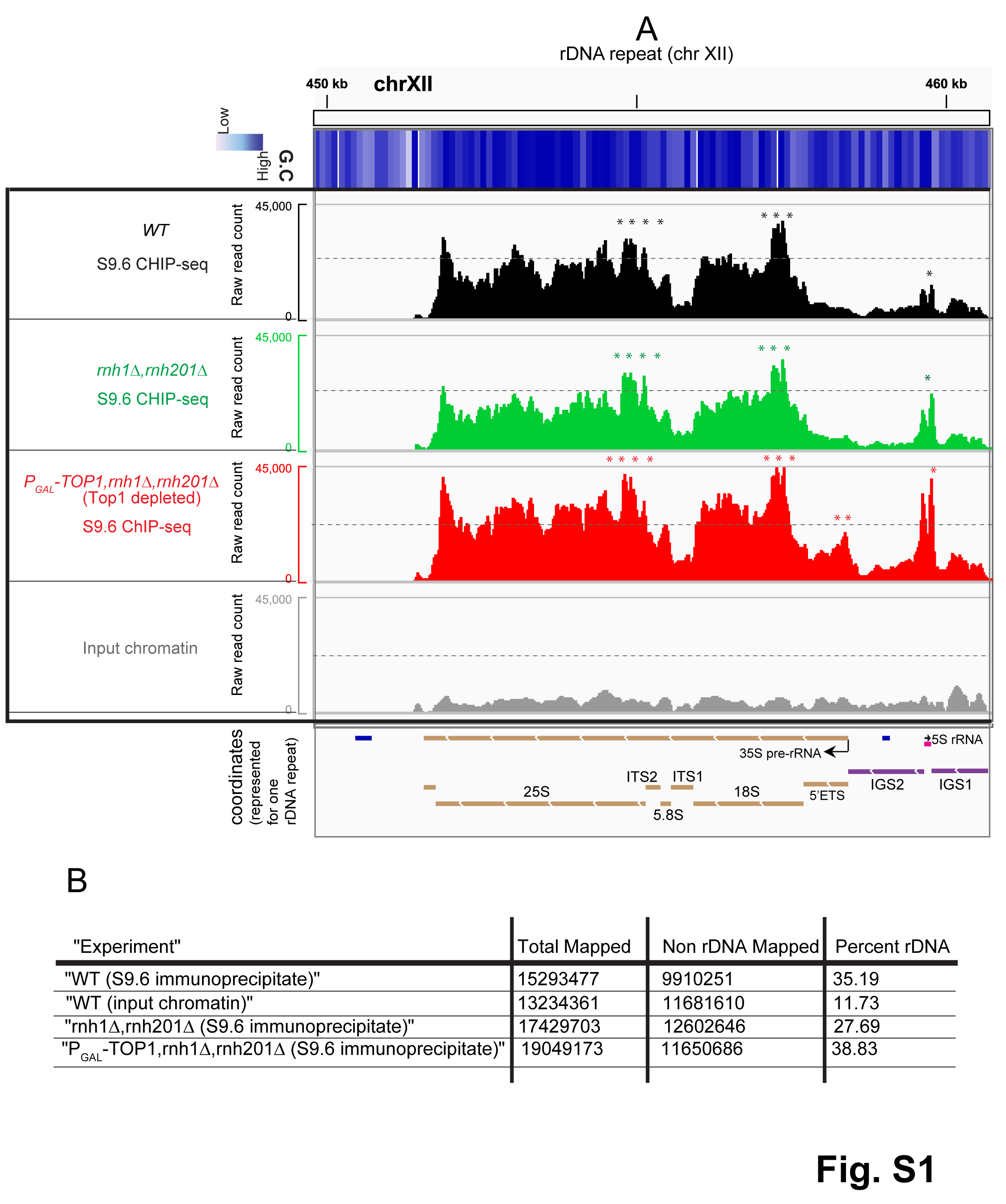

Supplement: Figure S1 — R-loops over the rDNA repeats are detected by S9.6 ChIP-seq. A: Analysis of R-loops by ChIP-seq using antibody S9.6 in the wild-type strain BY4741 (WT) and double mutant rnh1Δ rnh201Δ, and in triple mutant PGAL-TOP1 rnh1Δ rnh201Δ depleted of Top1 for 6 h at 30°C. Also shown is the input chromatin profile of the wild-type strain. Raw read counts were not corrected by the number of rDNA repeats. G+C content of the DNA sequence was calculated for 100 bp windows and is depicted as a blue intensity. Shown below the profiles is a diagram of one rDNA repeat which comprises the 35S rDNA gene, transcribed by Pol I in to the 35S pre-rRNA which is processed to 18S, 5.8S, and 25S rRNAs. 35S genes are flanked by the intergenic spacers IGS1 and IGS2 and the 5S rDNA gene, transcribed by Pol III. The direction of transcription is indicated by a tailed arrow. Chr = chromosome. Prominent R-loop peaks discussed in the text are highlighted by stars. ETS = external transcribed spacer. ITS = internal transcribed spacer. Profiles were generated using Integrative Genomics Viewer [100]. B: Relative recovery of rDNA sequences in the “input chromatin” and “S9.6-immunoprecipitates.” (TIF) [file pgen.1004716.s001.tif]

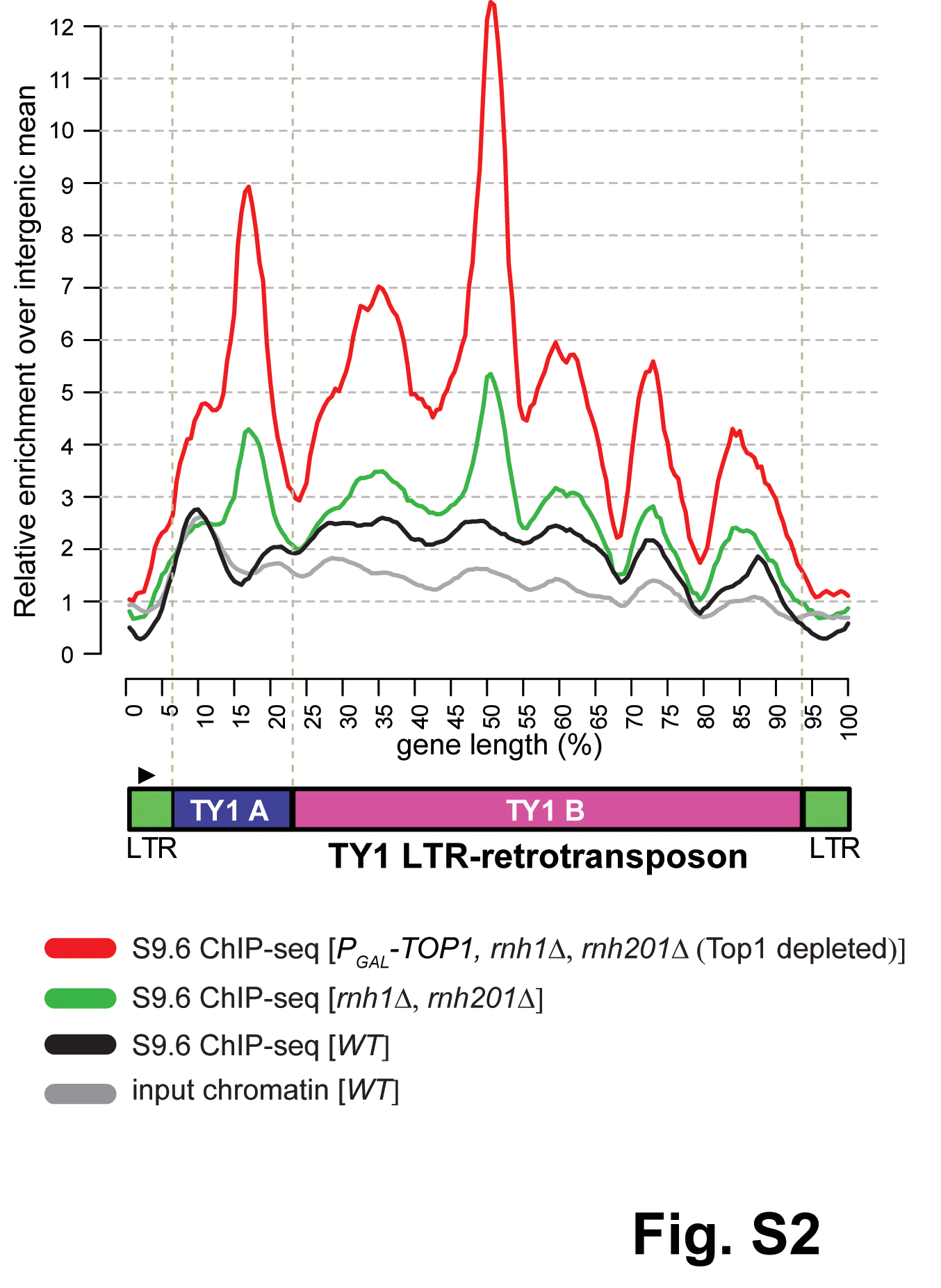

Supplement: Figure S2 — Average profiles of RNA-DNA hybrids over all Ty1 elements. Median S9.6 ChIP-seq profiles of RNA-DNA hybrids over the 31 Ty1 elements in strains WT (BY4741) and double mutant rnh1Δ rnh201Δ, and in triple mutant PGAL-TOP1 rnh1Δ rnh201Δ depleted of Top1 for 6 h at 30°C. Median profiles of control input chromatin from WT are also shown. The y-axis represents the relative enrichment of reads where values >1 are above the background level of sequencing (i.e. general intergenic mean, see Materials and Methods). (TIF) [file pgen.1004716.s002.tif]

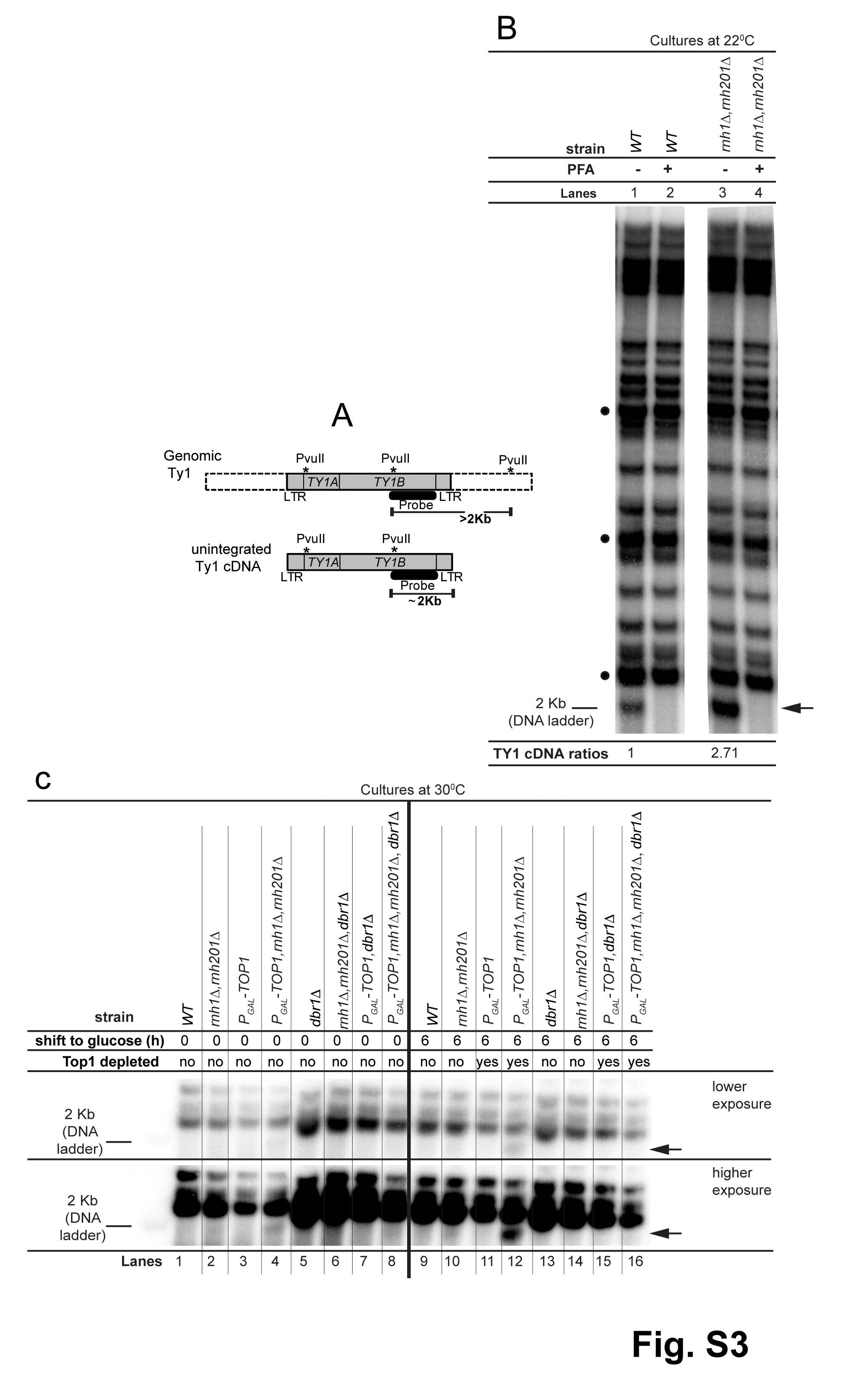

Supplement: Figure S3 — TY1 cDNAs accumulate in mutants lacking cellular RNase H or also depleted of Top1. A: Diagrams of unintegrated Ty1 cDNA and a genomic Ty1 element, indicating the location of the TY1B hybridization probe (filled black rectangle) and relevant PvuII cleavage sites. Probe TY1B detects an ∼2 Kb PvuII DNA fragment of unintegrated Ty1 cDNA and variably sized PvuII DNA fragments >2 Kb containing the junction of Ty1 elements with chromosomal DNA at different locations in the genome. B: Southern analyses of Ty1 cDNA from strain WT (BY4741) and double mutant rnh1Δ rnh201Δ, grown at 22°C in YEPD medium (glucose 2%) until saturation, in the absence or presence of 600 µg/ml of phosphonoformic acid (PFA), which is an inhibitor of TY1 RT [39]. The ratio of the ∼2 Kb Ty1 cDNA was determined by normalising the intensity of the Ty1 cDNA band relative to the average intensities of 3 genomic Ty1 junction bands (filled black circles). Values were expressed relative to the wild-type (-PFA, lane 1) which was set to 1. TY1 cDNA band is indicated by a tailed arrow. Indicated to the left of the gel the migration position of a 2 Kb band from a DNA size ladder. C: Southern analyses of Ty1 cDNAs from strain WT (BY4741) and mutant strains double rnh1Δ rnh201Δ, single PGAL-TOP1, triple PGAL-TOP1 rnh1Δ rnh201Δ, single dbr1Δ, triple rnh1Δ rnh201Δ dbr1Δ, double PGAL-TOP1 dbr1Δ, and quadruple PGAL-TOP1 rnh1Δ rnh201Δ dbr1Δ. Cultures were grown at 30°C in medium containing galactose and sucrose (permissive for PGAL-TOP1 expression, lanes 1–8) and shifted for 6 h to medium containing glucose (non-permissive for PGAL-TOP1 expression, lanes 9–16). TY1 cDNA band is indicated by a tailed arrow. Indicated to the left of the gel the migration position of a 2 Kb band from a DNA size ladder. (TIF) [file pgen.1004716.s003.tif]

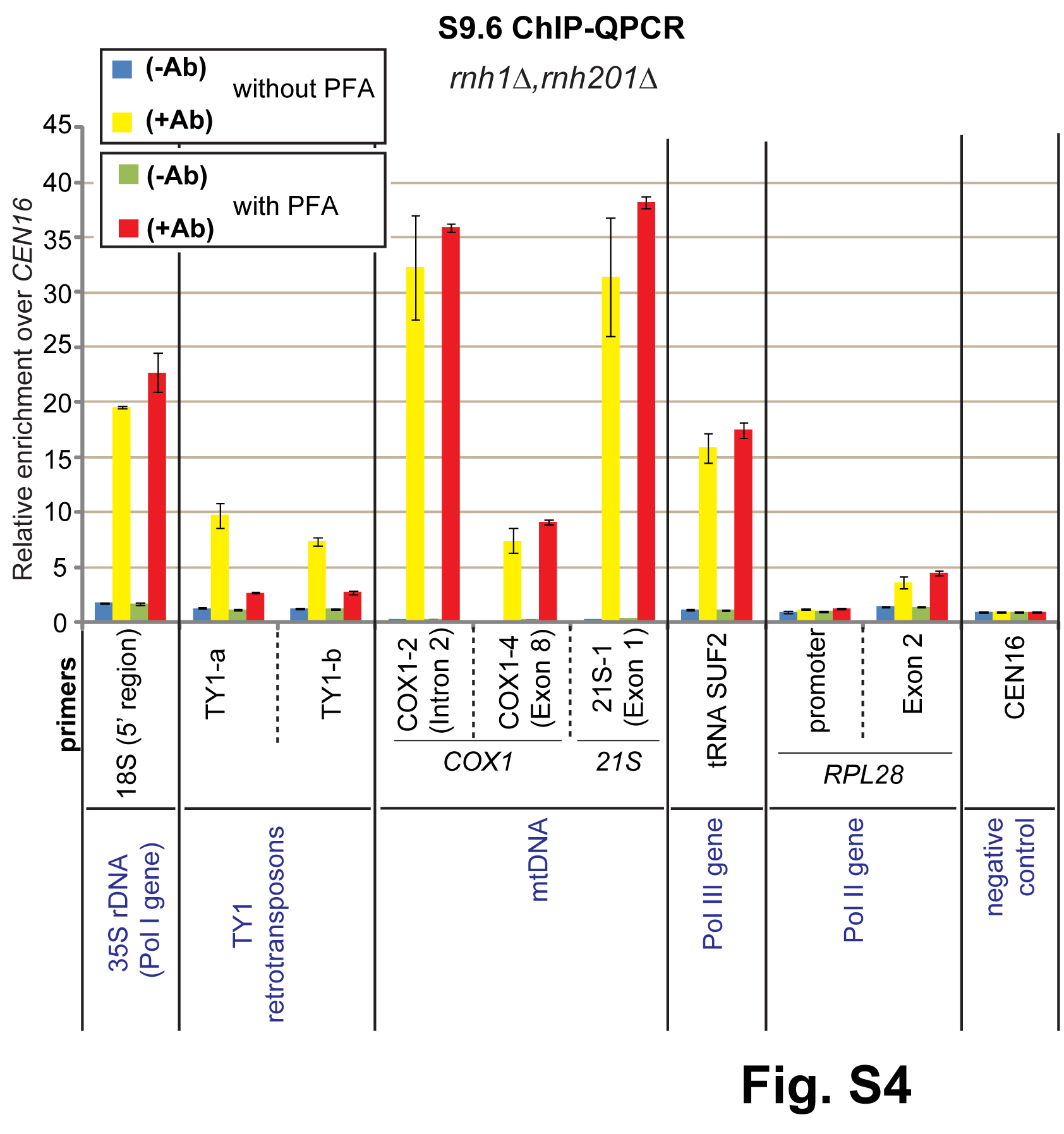

Supplement: Figure S4 — Reduced accumulation of RNA/DNA hybrids at Ty1 in the absence of RT activity. Double mutant rnh1Δ rnh201Δ was grown in YEPD medium (glucose 2%) at 22°C in the absence or presence of 600 µg/ml of phosphonoformic acid (PFA), which is an inhibitor of TY1 reverse transcriptase (RT) [39]. ChIPs were performed with no-antibody (−Ab) or antibody S9.6 (+Ab). The Pol I transcribed gene (18S rDNA), Ty1 retrotransposons, mtDNA transcription units (COX1 and 21S rDNA), Pol III gene tRNA SUF2, mRNA gene RPL28 and CEN16 were analyzed by Q-PCR as described in Fig. 1A. The mean of three independent experiments is shown with standard error. (TIF) [file pgen.1004716.s004.tif]

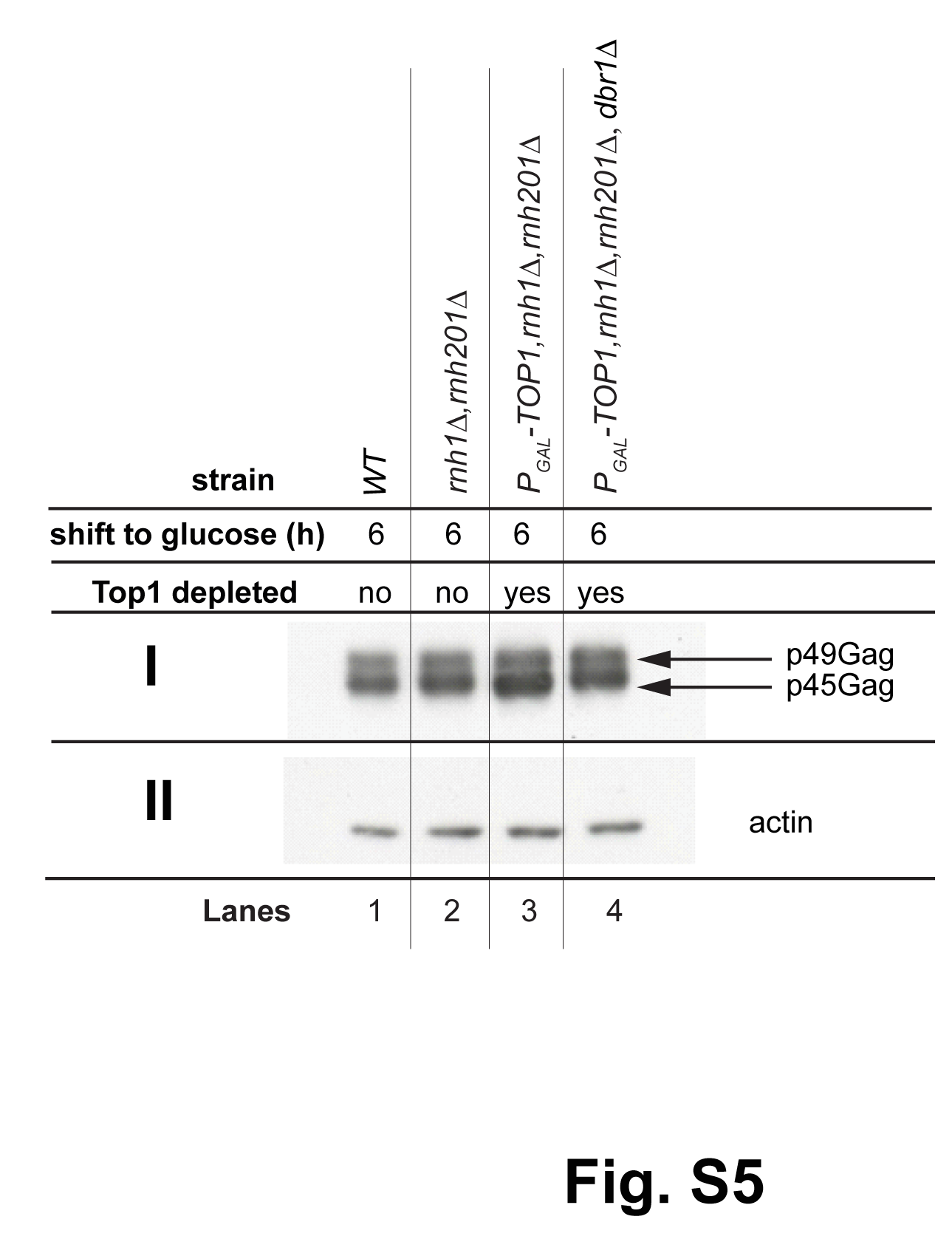

Supplement: Figure S5 — Gag proteins are slightly increased in mutants lacking both Top1 and cellular RNase H. Immunoblots of cellular homogenates from strain WT (BY4741) and mutant strains double rnh1Δ rnh201Δ, triple PGAL-TOP1 rnh1Δ rnh201Δ and quadruple PGAL-TOP1 rnh1Δ rnh201Δ dbr1Δ. Yeast cultures grown at 30°C were shifted from galactose- and sucrose- containing-medium to glucose medium and harvested at 6 h depletion of Top1. Panel I. Blot probed with antibody anti-Ty1 Gag. The Gag-p49 and processed-Gag-p45 bands each appear as doublets. Panel II. Blot probed with antibody anti-beta-actin. (TIF) [file pgen.1004716.s005.tif]

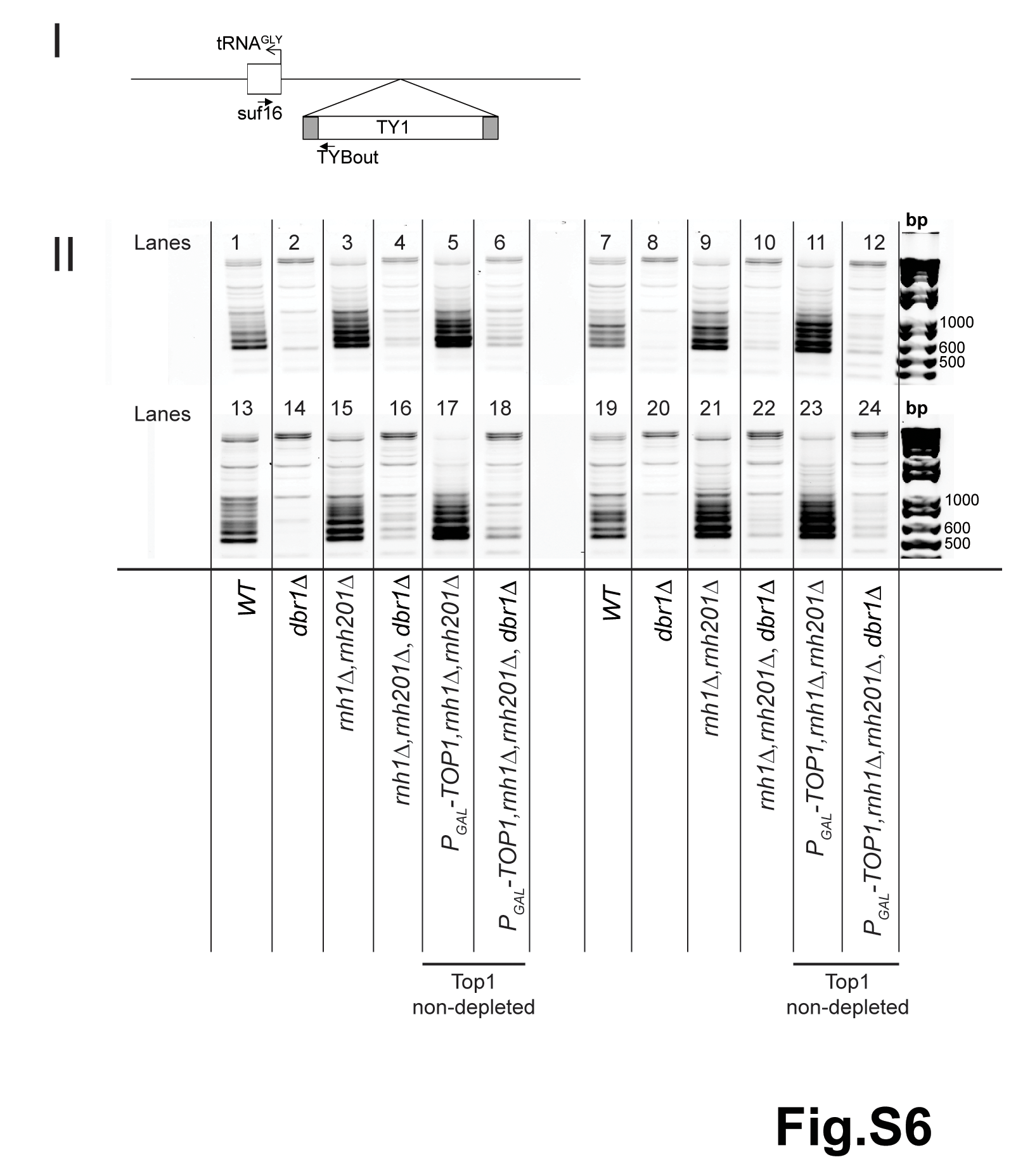

Supplement: Figure S6 — PCR analyses of integration of Ty1 at tRNAGLY in mutants lacking RNase H and/or Dbr1. Four independent isolates for each strain, WT (BY4741) and mutant strains single dbr1Δ, double rnh1Δ rnh201Δ, triple rnh1Δ rnh201Δ dbr1Δ, triple PGAL-TOP1 rnh1Δ rnh201Δ, and quadruple PGAL-TOP1 rnh1Δ rnh201Δ dbr1Δ, were grown until saturation at 18°C in medium containing both galactose and sucrose (permissive for PGAL-TOP1 expression). Panel I. See legend in Fig. 3D. Panel II. Representative examples of SYBR-stained gels are shown, revealing integration of Ty1 cDNA upstream of the 16 tRNAGLY gene loci. Shown to the right of the gels DNA ladders with lengths in base-pairs (bp). (TIF) [file pgen.1004716.s006.tif]

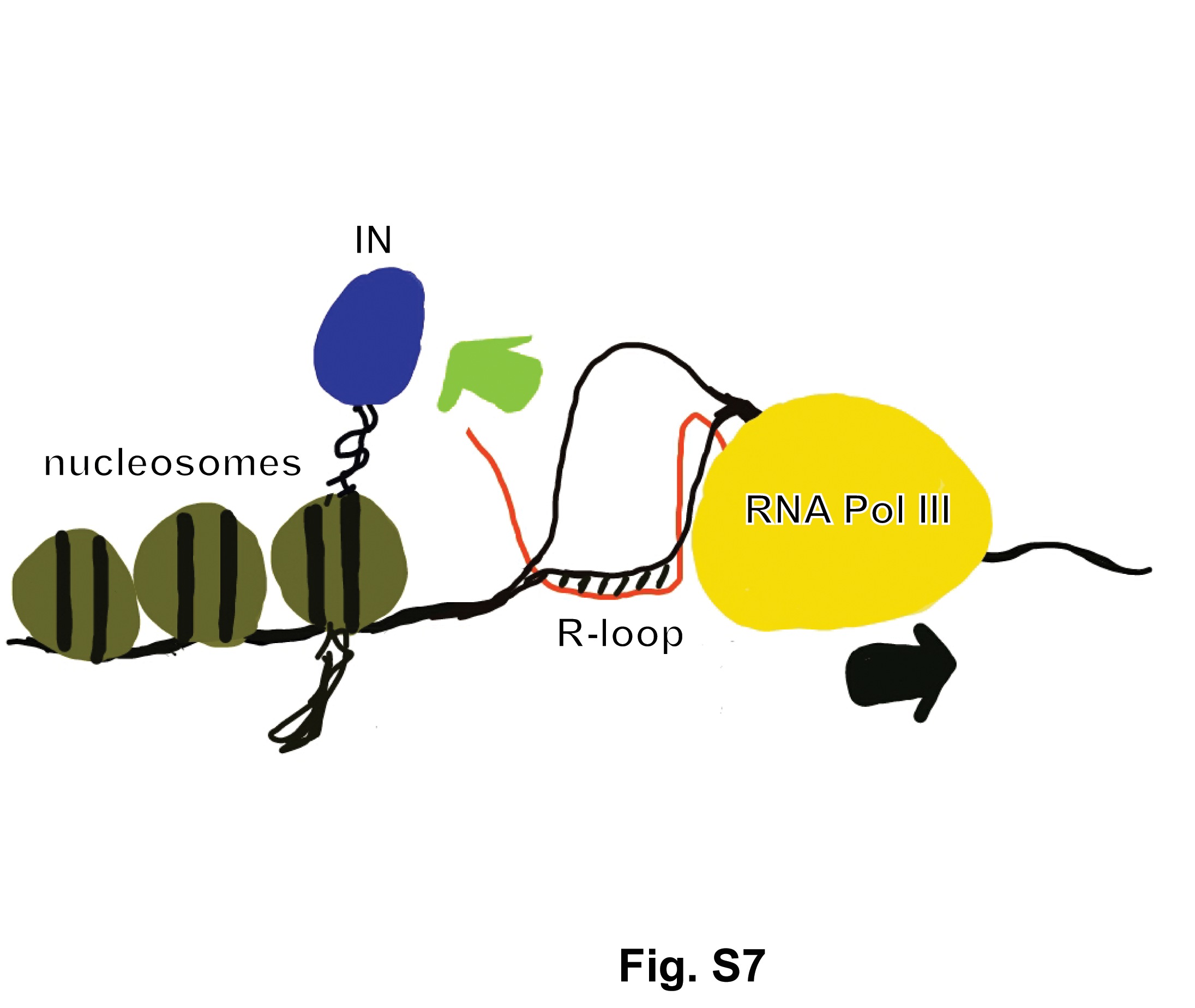

Supplement: Figure S7 — Model: Pol III-associated R-loops facilitate targeting of TY1 at 5′ flanking regions of tRNA genes. Ty1 integration upstream of tRNA genes is specifically targeted to the H2A/H2B interface of nucleosomal DNA in a ∼1 kb window [43], [44], [67]. The nascent transcript behind elongating Pol III can invade the DNA duplex and hybridize with the DNA template strand, generating a three-stranded R-loop structure, composed of an RNA-DNA duplex and an unpaired non-template DNA strand. We postulate that alterations in chromatin structure due to R-loop formation [102], [103] at Pol III genes, favor recruitment of the TY1 pre-integration complex formed by the integrase (IN) and the cDNA (green thick arrow = positive regulation). Black thick arrow = transcription direction. The diagram is not drawn to scale. (TIF) [file pgen.1004716.s007.tif]

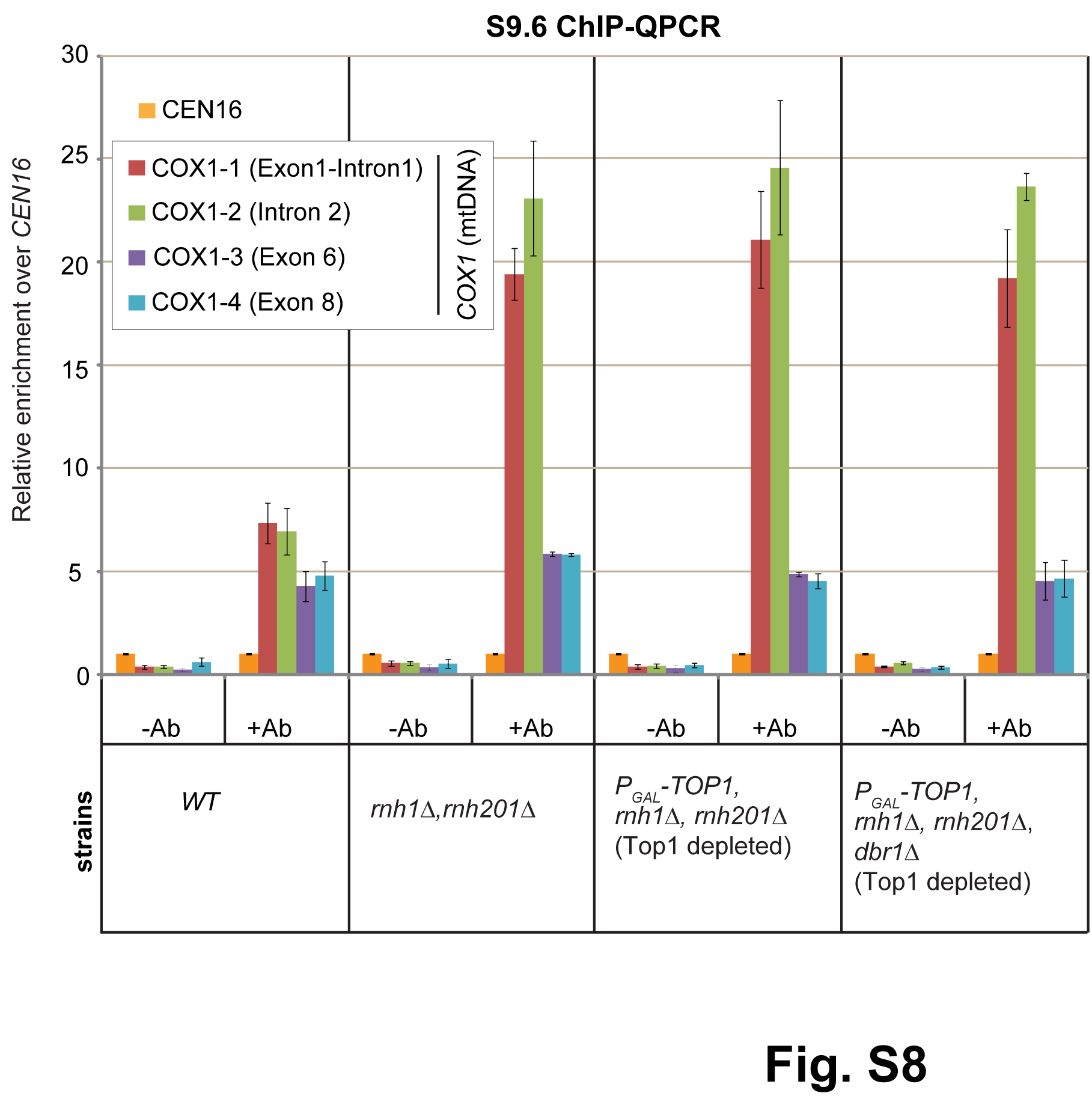

Supplement: Figure S8 — ChIP-QPCR of R-loops at mtDNA in mutants lacking both Top1 and cellular RNase H. ChIP samples using antibody S9.6 (same as in Fig. 3A) are from strains WT (BY4741) double mutant rnh1Δ rnh201Δ and from mutants triple PGAL-TOP1 rnh1Δ rnh201Δ and quadruple PGAL-TOP1 rnh1Δ rnh201Δ dbr1Δ depleted of Top1 for 6 h at 30°C. CEN16 and four different regions of COX1 gene were analysed by Q-PCR as described in Fig. 1A. Ab = antibody S9.6. (TIF) [file pgen.1004716.s008.tif]

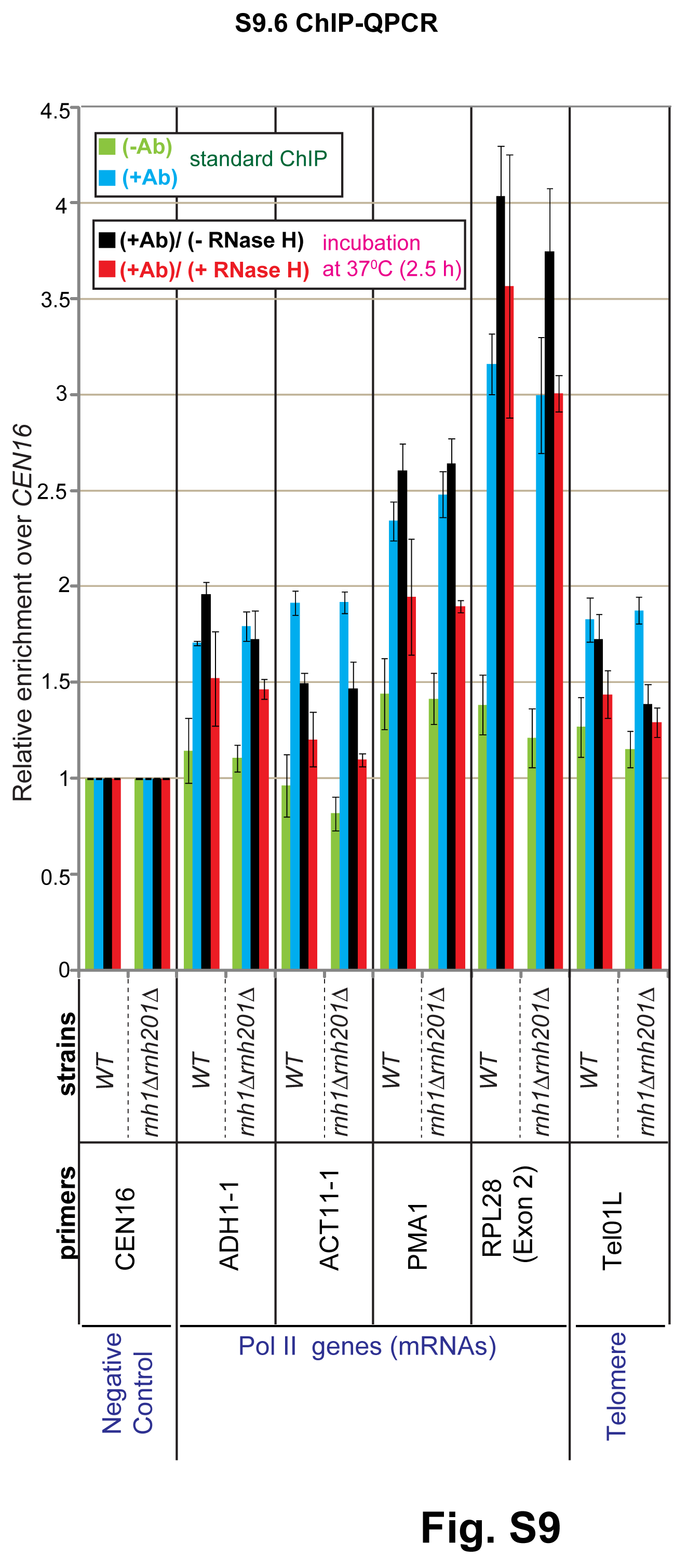

Supplement: Figure S9 — Formaldehyde-crosslinked R-loops associated with mRNA genes are slightly cleaved in vitro by recombinant RNase HI. ChIP samples are from strains WT (BY4741) and double mutant rnh1Δ rnh201Δ (same as in Fig. 1B) grown at 30°C in YEPD (glucose 2%). ChIPs were performed with no-antibody (−Ab) or antibody S9.6 (+Ab), or with antibody S9.6 but beads were further incubated for 2.5 h at 37°C in absence [(+Ab)/(−RNase H)] or presence [(+Ab)/(+RNase H)] of recombinant RNase HI (see Material and Methods). CEN16, the highly Pol II transcribed mRNA genes ADH1, ACT1, PMA1 and RPL28 (exon 2), and the telomeric region Tel01L, were analyzed by Q-PCR as described in Fig. 1A. (TIF) [file pgen.1004716.s009.tif]

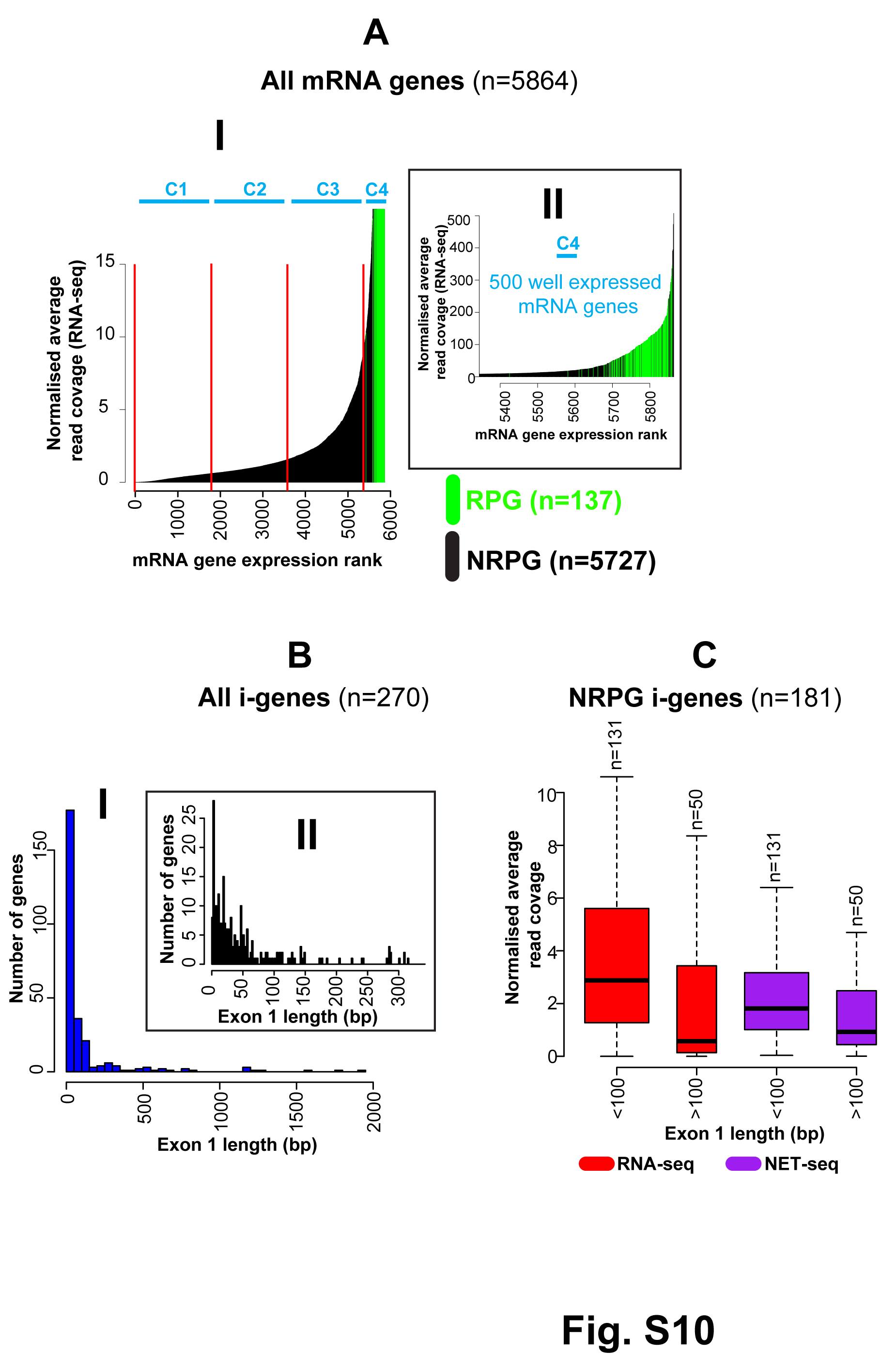

Supplement: Figure S10 — Expression of spliced genes with a “short-exon1” is generally higher than those with a “long-exon1.” A: Plot of RNA expression of yeast mRNA genes. Panel I. Raw transcriptome sequencing (RNA-seq) reads of exponentially growing wild-type strain BY4741 [101] were processed as described in Protocol S1. Normalised reads per base of exon of the 5864 protein-coding genes were plotted on the Y-axis. The top value of the Y-axis was arbitrarily set to 15. We clustered the mRNA genes into four groups of RNA expression, and indicated their boundaries by red vertical lines on the plot: C1 (low, n = 1788), C2 (medium-low, n = 1788), C3 (medium-high, n = 1788) and C4 (high, n = 500). The seventy four very-lowly expressed mRNA genes (subgroup “C1-0” in Fig. 5A) were included in group C1. The top ninety very-highly expressed mRNA genes (subgroup “C4-max” in Fig. 5A) were included in group C4. Ribosomal protein genes (RPG) and non-ribosomal protein genes (NRPG) are represented by a green or black vertical line, respectively. The plot region featuring the 500 highly expressed mRNA genes of group C4 including all the RPGs is magnified in panel II. n = number of genes. B: Plot of yeast mRNA intron-genes (i-genes) grouped according to the length of their first exon (exon 1) (panel I). The region of the plot featuring the i-genes with Exon 1 <300 bp is magnified in panel II: 87 RPGs (out of 89 i-genes) and 144 NRPGs (out of 181 i-genes) have an Exon 1 <300 bp. C: Boxplots of RNA-seq and Net-seq data from wild-type strain BY4741 [101] covering the 181 i-genes of NRPG (the 3 dubious open reading frames YDR535C, YLR202C and YOR318C were excluded from our analysis in Figs. 5, , S11, S13, S14), which were divided in to two groups based on the length of Exon1 (<100 and >100 bp). Normalised average read coverage of RNA-seq and NET-seq data (the number of reads per base of exon; see Protocol S1 and [101]) were calculated for each i-gene and the two Exon1-groups were represented as boxplots. Box- [file pgen.1004716.s010.tif]

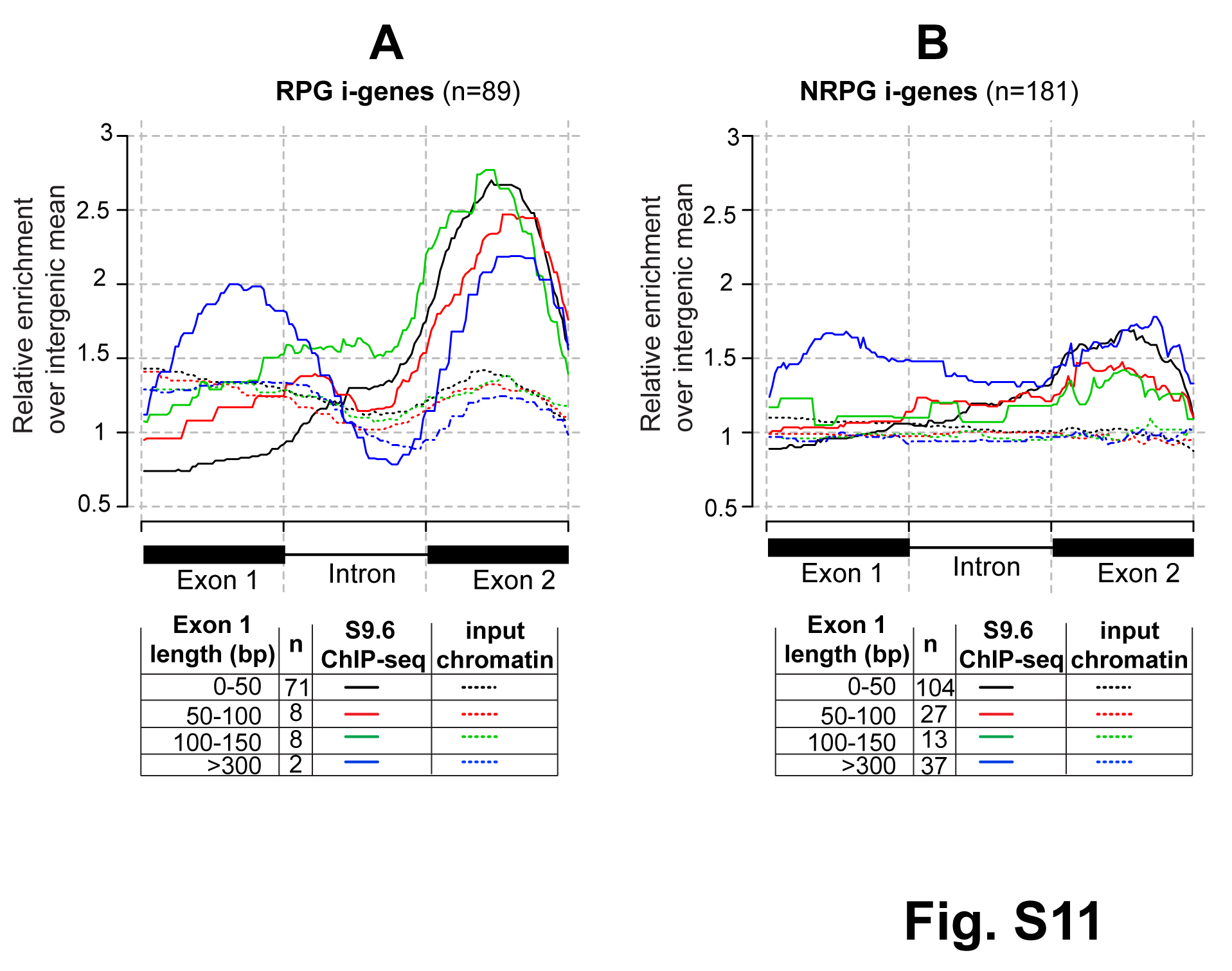

Supplement: Figure S11 — R-loop distribution over mRNA spliced genes according to the length of their first exon. Average profiles of S9.6 ChIP-seq and input chromatin of mRNA intron-genes (i-genes) in the wild-type strain (BY4741), grown at 30°C in YEPD medium (glucose 2%). Averaged reads were plotted on sequences encompassing Exon1-intron-Exon2 regions as described in Protocol S1. The 5′ end of Exon 1 is defined either as the AUG start codon, or 100 bp upstream of the 5′ splice site for genes with Exon 1 <100 pb (see also Protocol S1). The i-genes were split in to the ribosomal protein genes (RPG) (panel A) and the non-ribosomal-protein genes (NRPG) (panel B), and further segregated in to four sub-categories according to the length of their first exon (Exon 1): (0–50 bp), (50–100 bp), (100–150 bp) and (>300 bp). The y-axis represents the relative enrichment of reads where values>1 are above the background level of sequencing (i.e. general intergenic mean, see Materials and Methods). (TIF) [file pgen.1004716.s011.tif]

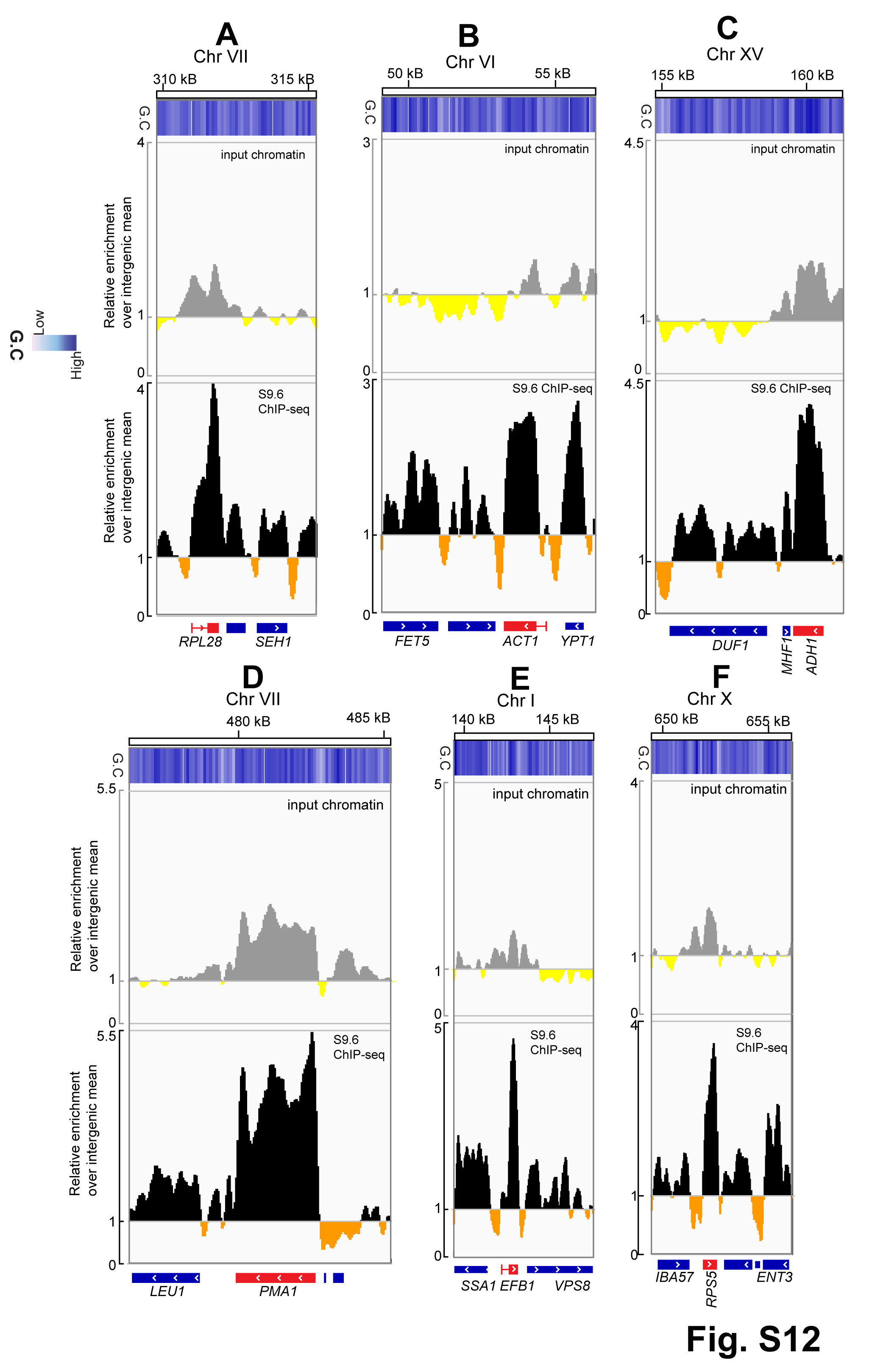

Supplement: Figure S12 — Examples of S9.6 ChIP-seq profiles of mRNA genes. Profiles of input chromatin and S9.6 ChIP-seq are for the wild-type strain (BY4741) grown at 30°C in YEPD medium (glucose 2%). Shown for spliced genes RPL28 (A), ACT1 (B) and EFB1 (E) and intronless genes SEH1 (A), FET5 and YPT1 (B), DUF1, MHF1 and ADH1 (C), LEU1 and PMA1 (D), SSA1 and VPS8 (E), and IBA57, RPS5 and ENT3 (F). The y-axis represents the relative enrichment of reads where values >1 are above the background level of sequencing (i.e. general intergenic mean, see Materials and Methods). G+C content of the DNA sequence was calculated for 100 bp windows and is depicted as a blue intensity. Shown below the profiles is a graphical representation of genomic features, with exon and intron sequences depicted as filled boxes and horizontal lines, respectively. The direction of transcription is indicated by a tailless arrow. Highly expressed genes are colored in red. Chr = chromosome. Profiles were generated using Integrative Genomics Viewer [100]. (TIF) [file pgen.1004716.s012.tif]

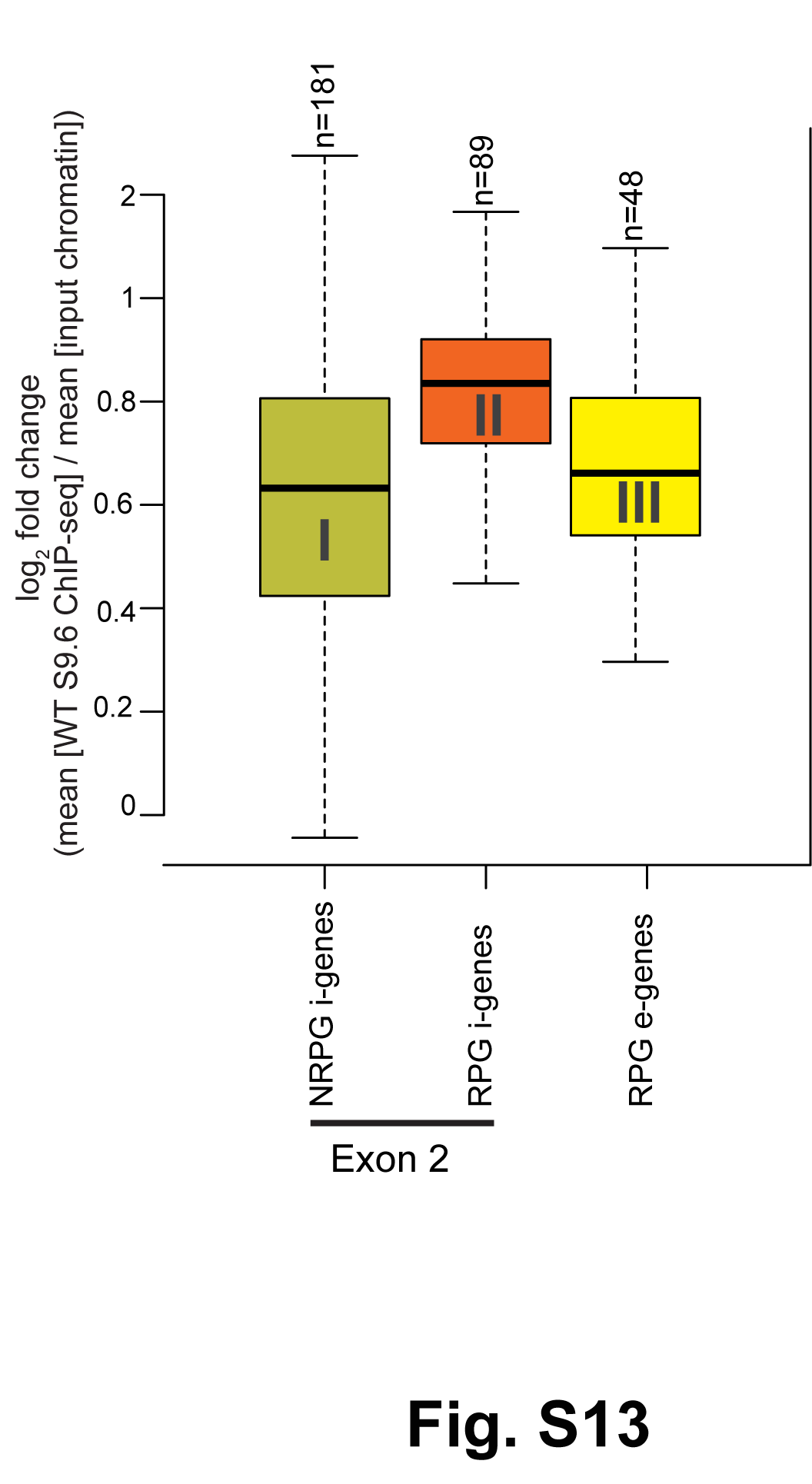

Supplement: Figure S13 — Comparison of R-loop distribution across Exon 2 of spliced-genes and across non-spliced ribosomal protein genes. Box plots of mean sequence read distribution of R-loops per gene across the second exon of the i-genes NRPG (I) and RPG (II), and the entire length of the e-genes RPG (III). Each box plot represents the log2 fold change of mean S9.6 ChIP-seq relative to input chromatin in the wild-type (BY4741), grown at 30°C in YEPD medium (glucose 2%). Box-plot representation shows median values (black line) +/−25% quartiles in the box and minimum/maximum distribution of the values (excluding outliers) in the whiskers (the regions above zero value on the Y-axis are enriched with R-loops; see also Fig. 5). n = number of genes. RPG = ribosomal protein genes. NRPG = non-ribosomal protein genes. i-genes = intron-containing genes. E-genes = intronless genes. We used a Kolmogorov-Smirnov test to determine whether levels of log2 fold change of mean sequence distribution differ significantly between box plots I and II (D = 0.449314048047675, p-value = 6.90119072999096e-11), and II and III (D = 0.39559925093633, p-value = 6.55907560322966e-05). (TIF) [file pgen.1004716.s013.tif]

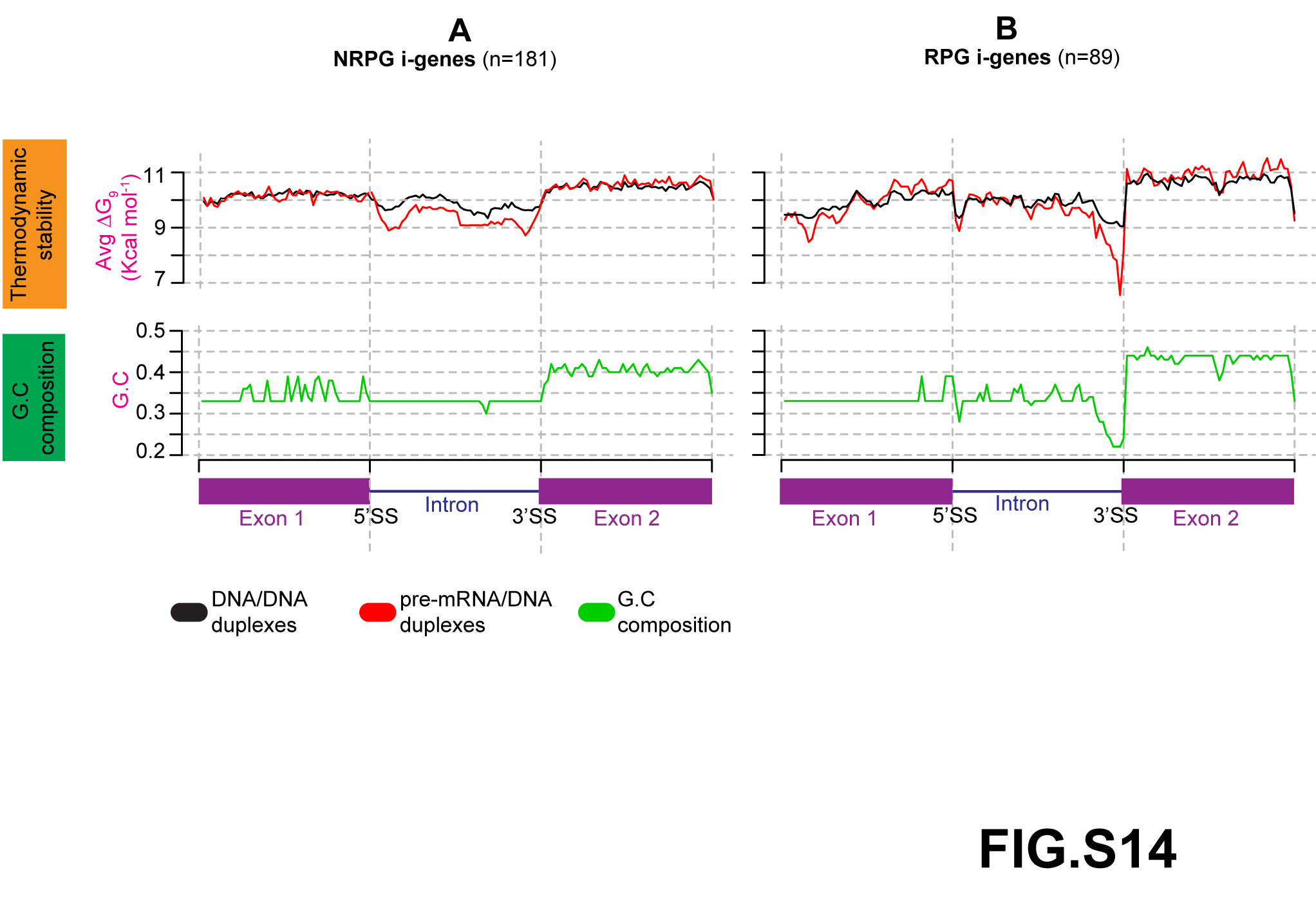

Supplement: Figure S14 — Thermodynamic helical stability of DNA/DNA and (pre-mRNA)/DNA duplexes of yeast mRNA intron-genes. The mRNA intron-genes (i-genes) were split in to the non-ribosomal-protein genes (NRPG) (A) and the ribosomal protein genes (RPG) (B). The thermodynamic helical stability of polynucleotide sequences (ΔG9 values of DNA/DNA and (pre-mRNA)/DNA duplexes) and the concentrations of G.C nucleotides were calculated for non-overlapping windows of 9 bases as described in Protocol S1 and [12]. Averaged values of ΔG9 and [G+C] nucleotides for the i-genes in each group were plotted on sequences encompassing Exon1-intron-Exon2 regions as described in Protocol S1. The 5′ end of Exon 1 is defined either as the AUG start codon, or 100 bp upstream of the 5′ splice site for genes with Exon 1 <100 pb (see also Protocol S1). n = number of genes. 5′SS = 5′ splice site. 3′SS = 3′ splice site. (TIF) [file pgen.1004716.s014.tif]

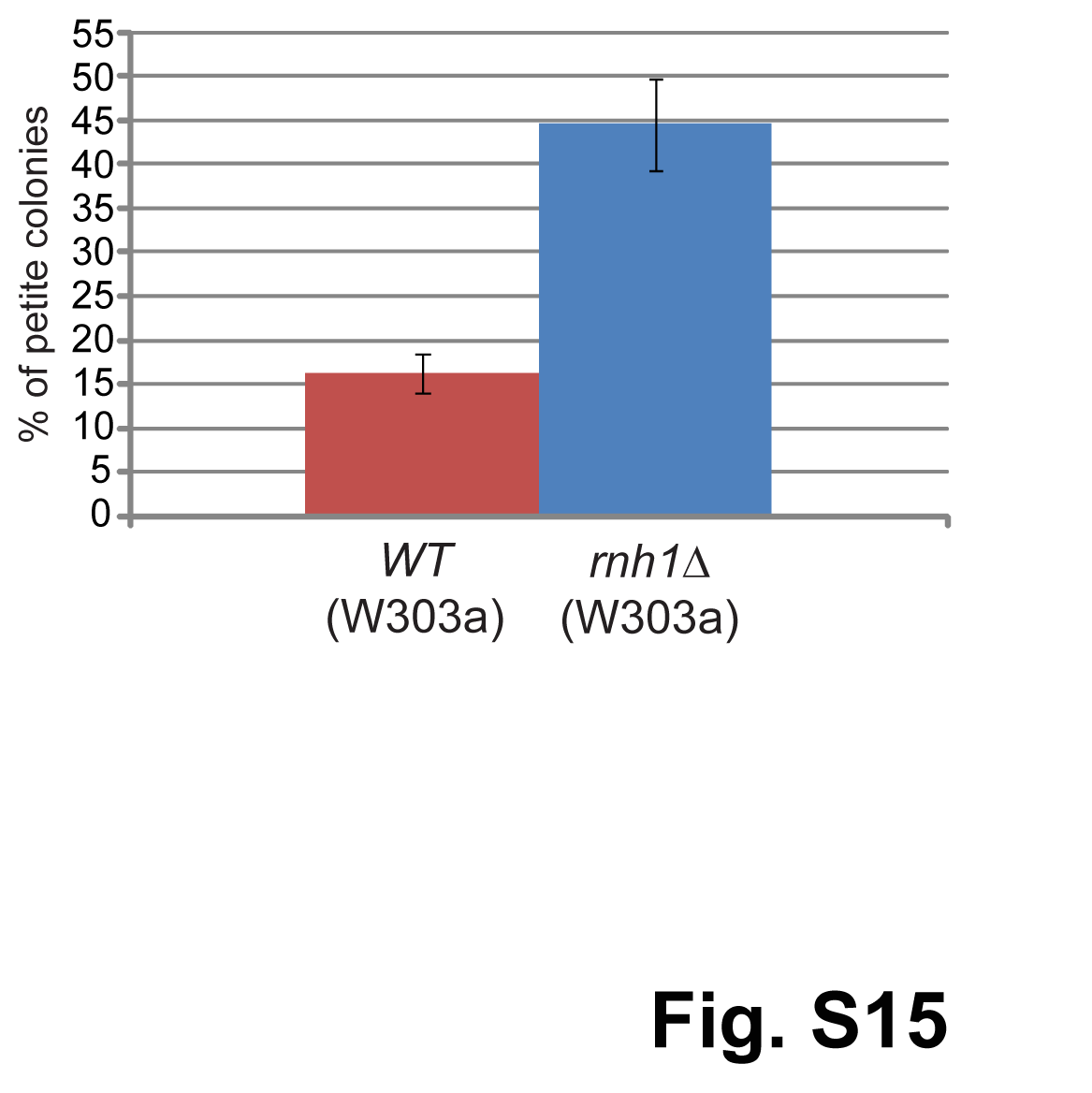

Supplement: Figure S15 — Petite frequency is higher in yeast rnh1Δ mutants than in isogenic wild-type W303a. Strains wild-type W303a and mutant rnh1Δ were grown at 30°C in YEP medium containing 3% glycerol. After four days cells were washed with sterile water, diluted appropriately, plated onto YEP medium containing 2% glucose and incubated at 30°C. One feature of ‘petite’ ade2− cells is to have a small size and the inability to convert an intermediate in the adenine biosynthesis pathway (AIR) into a red pigment due to impaired respiratory functions (e.g. see [104]). Conversely, ‘grande’ ade2− cells have a relatively large size and accumulate a red pigment due to functional mitochondria. For each strain a total of 8000 colonies were counted and petite/white and grande/red colonies were scored. Values are means of ten independent isolates with standard errors. (TIF) [file pgen.1004716.s015.tif]

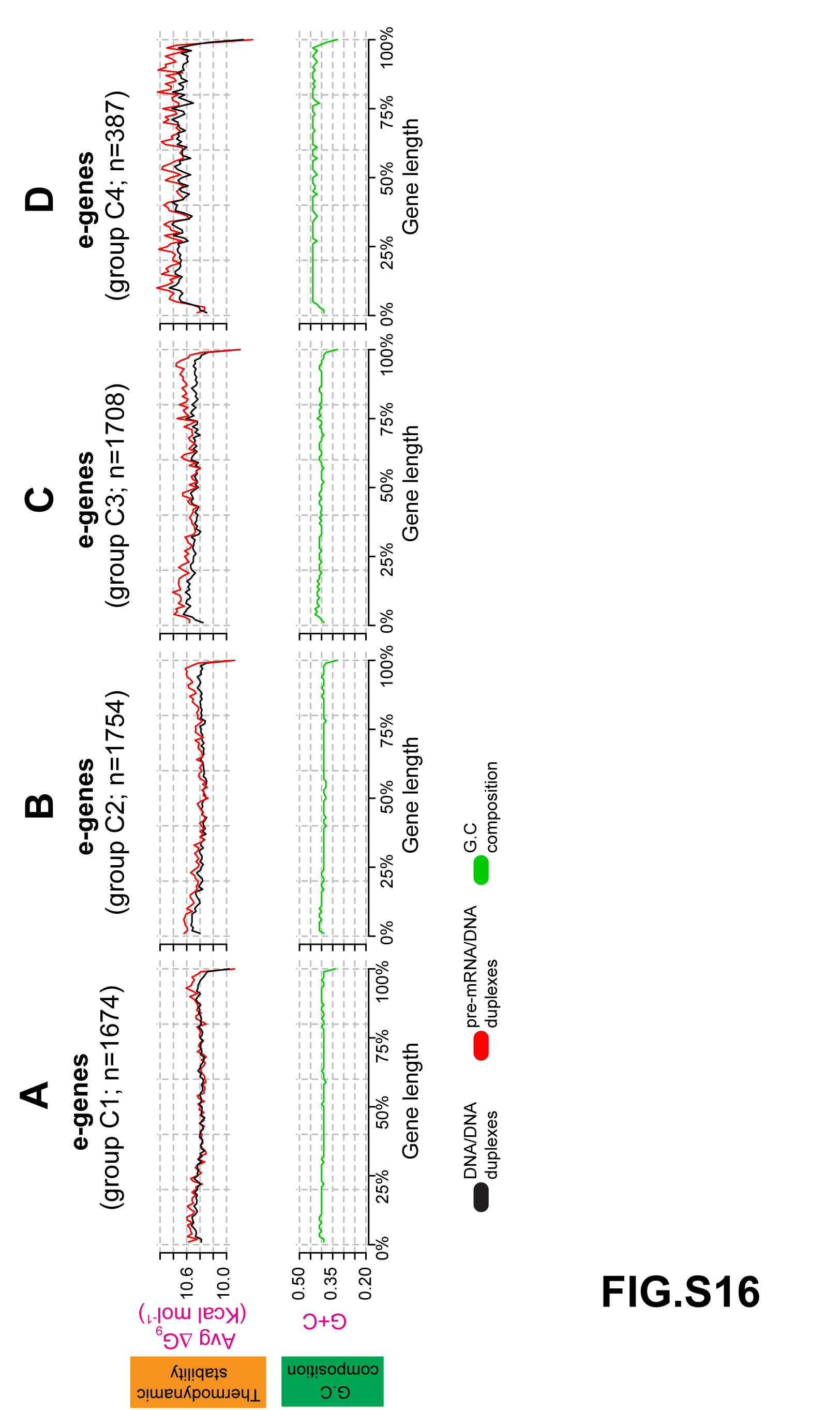

Supplement: Figure S16 — Thermodynamic helical stability of DNA/DNA and (pre-mRNA)/DNA duplexes of yeast mRNA intronless genes. The mRNA intronless genes (e-genes) were split in to four groups according to their mRNA expression: panel A (group C1, low expression, n = 1674), panel B (group C2, medium-low expression, n = 1754), panel C (group C3, medium-high expression, n = 1708) and panel D (group C4, high expression, n = 387). For ΔG9 values of DNA/DNA and (pre-mRNA)/DNA duplexes and [G+C] nucleotides see Fig. S14 and Protocol S1. Averaged values of ΔG9 and [G+C] for the e-genes in each group were plotted on sequences encompassing the entire length of the gene (0–100%). n = number of genes. (TIF) [file pgen.1004716.s016.tif]
