# Supplementary material for: Genome-Wide Distribution of RNA-DNA Hybrids Identifies RNase H Targets in tRNA Genes, Retrotransposons and Mitochondria
Source: PLoS Genet. 2014 Oct 30;10(10):e1004716. doi: 10.1371/journal.pgen.1004716 (PMC4214602; doi:10.1371/journal.pgen.1004716)
Supplement: Table S1 — List of strains and plasmids. A: Strains. B: Plasmids. (DOC) [file pgen.1004716.s017.doc]

**Table S1: List of strains and plasmids**

| **A: Strains** |  |  |
| --- | --- | --- |
| **Name** | **Genotype** | **Reference** |
| BY4741 | *MATa his3Δ1 leu2Δ0 met15Δ0 ura3Δ0* | Invitrogen |
| YAEH354 | BY4741 but *rnh1Δ* (KanMx6) | Invitrogen |
| YAEH355 | BY4741 but *rnh201Δ* (KanMx6) | Invitrogen |
| YAEH255 | BY4741 but *rnh1Δ* (KanMx6) *rnh201Δ* (NatMx6) | [23] |
| YAEH271 | BY4741 but *P_GAL1_-3HA-TOP1* (KanMx6) | [23] |
| YAEH275 | BY4741 but *P_GAL1_-3HA-TOP1* (KanMx6) *rnh201Δ* (NatMx6) *rnh1Δ* (HphMx6) | [23] |
| YAEH316 | YAEH271 but *P_GAL1_-3HA-TOP2* (HIS3Mx6) | [23] |
| YAEH317 | YAEH275 but *P_GAL1_-3HA-TOP2* (HIS3Mx6) | [23] |
| YAEH394 | BY4741 but *dbr1Δ* (His3Mx6) | This study |
| YAEH395 | YAEH271 but *dbr1Δ* (His3Mx6) | This study |
| YAEH396 | YAEH275 but *dbr1Δ* (His3Mx6) | This study |
| YAEH431 | YAEH255 but *dbr1Δ* (His3Mx6) | This study |
| YAEH419 | JC3212= BY4741 but *TY1his3AI-[Δ1]-3114* | [41] |
| YAEH424 | YAEH419 but *rnh1Δ* (HphMx6) | This study |
| YAEH425 | YAEH419 but *rnh201Δ* (NatMx6) | This study |
| YAEH426 | YAEH419 but *rnh1Δ* (HphMx6) *rnh201Δ* (NatMx6) | This study |
| YAEH438 | YAEH419 but *P_GAL1_-3HA-TOP1* (KanMx6) | This study |
| YAEH439 | YAEH426 but *P_GAL1_-3HA-TOP1* (KanMx6) | This study |
| YAEH480 | YAEH419 + vector | This study |
| YAEH483 | YAEH419 + p*RNH201-FLAG2* | This study |
| YAEH484 | YAEH419 + p*rnh201::G42S-FLAG2* | This study |
| YAEH481 | YAEH426 + vector | This study |
| YAEH485 | YAEH426 + p*RNH201-FLAG2* | This study |
| YAEH486 | YAEH426 + p*rnh201::G42S-FLAG2* | This study |
| YAEH482 | YAEH438 + vector (ycplac111) | This study |
| YAEH475 | YAEH439 + vector (ycplac111) | This study |
| YAEH476 | YAEH439 + p*RNH201-FLAG2* | This study |
| YAEH478 | YAEH439 + p*rnh201::G42S-FLAG2* | This study |
| W303a | *MAT a leu2-3,112 trp1-1 can1-100 ura3-1 ade2-1 his3-11,15* | R. Rothstein |
| YAEH519 | W303a but *rnh1Δ* (KanMx6) | This study |
| **B: Plasmids** |  |  |
| **Name** | **Description** | **Reference** |
| vector | ycplac111 | [42] |
| p*RNH201-FLAG2* | wild-type *RNH201* gene expressed under its native promoter | [42] |
| p*rnh201::G42S-FLAG2* | AGS-related mutant *rnh201::G42S-FLAG2* expressed under its native promoter | [42] |
| pGTyH3 | Ty1-H3 element expressed under *P_GAL_* promoter | **a** |

**^a^** Boeke JD, Eichinger D, Castrillon D, Fink GR (1988) The Saccharomyces cerevisiae genome contains functional and nonfunctional copies of transposon Ty1. Mol Cell Biol 8: 1432-1442.
