# Supplementary material for: Genome-Wide Distribution of RNA-DNA Hybrids Identifies RNase H Targets in tRNA Genes, Retrotransposons and Mitochondria
Source: PLoS Genet. 2014 Oct 30;10(10):e1004716. doi: 10.1371/journal.pgen.1004716 (PMC4214602; doi:10.1371/journal.pgen.1004716)
Supplement: Table S2 — Oligonucleotides used in Northern and Southern analysis. A: Oligonucleotides for Northern analysis of tRNAs and rRNAs. B: Oligonucleotides for Southern analysis of Ty1 cDNAs. (DOC) [file pgen.1004716.s018.doc]

**Table S2: Oligonucleotides used in Northern and Southern analysis**

| **A: Oligonucleotides for Northern analysis of tRNAs and rRNAs** | | |
| --- | --- | --- |
| **Name** | **Sequence^a^** | **Specifications** |
| tRNA_2_^LYS^ | AAAGTAAAGAACTCCTCATAG**^b^** | Primer hybridizes to the region encompassing the 3’-mature-end and  3’-flank of tRNAs *tK(UUU)K*, *tK(UUU)L* and *tK(UUU)P*. It hybridizes specifically to both precursor and mature species. |
| tRNA^TRP^ | AACCTGCAACCCTTCGA**^b^** | Primer hybridizes to exon 2 of intron-containing tRNAs *tW(CCA)G1*, *tW(CCA)G2*, *tW(CCA)J*, *tW(CCA)K*, *tW(CCA)M* and *tW(CCA)P*. |
| tRNA_3_^LEU^ | GCATCTTACGATACCTG**^b^** | Primer hybridizes to exon 2 of intron-containing tRNAs *tL(CAA)A*, *tL(CAA)C, tL(CAA)D*, *tL(CAA)G1*, *tL(CAA)G2*, *tL(CAA)G3*, *tL(CAA)K*, *tL(CAA)L*, *tL(CAA)M* and *tL(CAA)N*. |
| tRNA_i_^MET^ | GTTTCGATCCGAGGACATCAG**^c^** | Primer hybridizes to tRNAs *tM(CAU)C*, *tM(CAU)D*, *tM(CAU)E*, *tM(CAU)J1*, *tM(CAU)J2*, *tM(CAU)J3*, *tM(CAU)M*, *tM(CAU)O1*, *tM(CAU)O2* and *tM(CAU)P*. tRNA precursors correspond to the short (*IMT1* + *IMT4*) and long (*IMT2*+ *IMT3*) forms of initiator tRNA-Met. |
| 5S rRNA | CTACTCGGTCAGGCTC |  |
| **B: Oligonucleotides for Southern analysis of Ty1 cDNAs** | | |
| **Name** | **Sequence^a^** | **Specifications** |
| Ty1cDNA-F | gatacgatgaggcaatcacc**^d^** | Primer pairs for the synthesis of PCR templates (encompassing coordinates 3992 to 5432 in the *TYB* sequence of Ty1-H3**^e^**),  which were used for the synthesis of DNA Random priming probes  to detect the ~2Kb PvuII fragment of the unincorporated Ty1 cDNA**^d^**). |
| Ty1cDNA-R | ggcacaaaggcaatgagact**^d^** |

**^a^** Oligonucleotides are indicated (5’-3’)

**^b^** Kufel J, Tollervey D (2003) 3'-processing of yeast tRNATrp precedes 5'-processing. RNA 9: 202-208.

**^c^** Karkusiewicz I, Turowski TW, Graczyk D, Towpik J, Dhungel N, et al. (2011) Maf1 protein, repressor of RNA polymerase III, indirectly affects tRNA processing. J Biol Chem 286: 39478-39488.

**^d^** Todeschini AL, Morillon A, Springer M, Lesage P (2005) Severe adenine starvation activates Ty1 transcription and retrotransposition in Saccharomyces cerevisiae. Mol Cell Biol 25: 7459-7472.

**^e^** Boeke JD, Eichinger D, Castrillon D, Fink GR (1988) The Saccharomyces cerevisiae genome contains functional and nonfunctional copies of transposon Ty1. Mol Cell Biol 8: 1432-1442.
