# Supplementary material for: Genome-Wide Distribution of RNA-DNA Hybrids Identifies RNase H Targets in tRNA Genes, Retrotransposons and Mitochondria
Source: PLoS Genet. 2014 Oct 30;10(10):e1004716. doi: 10.1371/journal.pgen.1004716 (PMC4214602; doi:10.1371/journal.pgen.1004716)
Supplement: Table S3 — Oligonucleotides used in ChIP-QPCR, PCR and ChIP-seq analysis. A: ChIP-QPCR primers. B: Standard PCR primers. C: ChIP-seq primers. (DOC) [file pgen.1004716.s019.doc]

**Table S3: Oligonucleotides used for ChIP-QPCR, standard PCR and ChIP-seq analysis**

| **A: ChIP-QPCR primers** |  |  |
| --- | --- | --- |
| **Name** | **Sequence^a^** | **[Name of amplified region as designated in manuscript] and specifications** |
| igs2-0-F | CCCTCCCATTACAAACTAAA | [Promoter]  35S rRNA transcribed by  RNA Pol I |
| igs2-0-R | GGCGAGAAATACGTAGTTAAG |
| 35S-F | CCACGATGAGACTGTTCAGG | [5’ETS(1)]  35S rRNA transcribed by  RNA Pol I |
| 35S-R | GTCGCTAGGTGATCGTCAGA |
| 5’ETS-F | AACAGCTGAAATTCCAGAAA | [5’ETS(2)]  35S rRNA transcribed by  RNA Pol I |
| 5’ETS-R | CTATGGTATGGTGACGGAGT |
| 1-18S2-F | TCCAATTGTTCCTCGTTAAG | [18S 5’region)]  35S rRNA transcribed by  RNA Pol I |
| 1-18S2-R | ATTCAGGGAGGTAGTGACAA |
| 25S3-F | TTACACCCAAACACTCGCAT | [25S]  35S rRNA transcribed by  RNA Pol I |
| 25S3-R | GACTGAGGACTGCGACGTAA |
| 5S rDNA-F | ACCTGCGTTTCCGTTAACT | [5S rDNA]  5S rRNA transcribed by  RNA Pol III |
| 5S rDNA-R | AGTTGATCGGACGGGAAAC |
| SCR1-F2 | TGTCCCGGCTATAATAAATC | [SCR1]  RNA scR1 transcribed by  RNA Pol III |
| SCR1-R2 | CCTAAGGACCCAGAACTACC |
| SNR6-F1 | TCATCGAGTGAAGTATCGTG | [SNR6]  Small nuclear RNA U6 (SNR6)  transcribed by RNA Pol III |
| SNR6-R1 | ATAGCAAAGGCTTAGGTTCG |
| tQ(UUG)L-F1 | GAATGACGTATGCTGCTTT | [tRNA tQ(UUG)L]  tRNA transcribed by RNA Pol III |
| tQ(UUG)L-R1 | TGGATTTAAACTTCTCACAGG |
| tS(GCU)F-F1 | CAAACCAAGAGATTCTGTCC | [tRNA tS(GCU)F]  tRNA transcribed by RNA Pol III |
| tS(GCU)F-R1 | AAGAAATGCGTCACAGACA |
| tP(AGG)C-F3 | ATAGGGCTGTATCCCAGACT | [tRNA SUF2]  tRNA transcribed by RNA Pol III |
| tP(AGG)C-R3 | caccctaagcgagaatcata |
| Ty1-a-F | TCGATGAAGACTTAAACCGT | [ty1-a]  Ty1 retrotransposon: region encompassing 3010 to 3334 in the *TYB* sequence of *YGRWTY1-1* |
| Ty1-a-R | TGGATTCTTGGATCTCATTT |
| Ty1-b-F | ACACCTCCGTCAACTCATAC | [ty1-b]  Ty1 retrotransposon: region encompassing 3010 to 3334 in the *TYB* sequence of *YGRWTY1-1* |
| Ty1-b-R | TCTCGATATTGGAAATTTGG |
| COX1-F1 | TATGGCAGGAACAGCAATGT | [COX1-1 (Exon 1-Intron1)]  Region encompassing Exon1-Intron1 in mitochondrial gene *COX1/Q0045*,  which has 8 exons and 7 introns. |
| COX1-R1 | ATGCTAGACGCATCAACGAA |
| COX1-F2 | GGGATTGTGATTCATGCTTATG | [COX1-2 (Intron 2)]  Intron 2 in mitochondrial gene *COX1/Q0045*,  which has 8 exons and 7 introns. |
| COX1-R2 | CGGACAATCCCGTATTTCTT |
| COX1-F3 | AGCTCTAATCCATGGTGGTTC | [COX1-3 (Exon 6)]  Exon 6 in mitochondrial gene *COX1/Q0045*,  which has 8 exons and 7 introns |
| COX1-R3 | TCGTGGAATGCTACATCTAATGA |
| COX1-F4 | GGTATGCCTAGAAGAATTCCTGA | [COX1-4 (Exon 8)]  Exon 8 in mitochondrial gene *COX1/Q0045*,  which has 8 exons and 7 introns |
| COX1-R4 | TGAATGAACCAATAGAAGCGA |
| 21S-F1 | CGAAAGCAAACGATCTAACT | [21S1 (Exon 1)]  Exon 1 in mitochondrial gene  *21S rDNA*,  which has 2 exons and 1 intron |
| 21S-R1 | AGCTATCTGCAAACCAGATT |
| 21S-F2 | TTCCTTGGCCTATAATTGAG | [21S1 (Exon 1)]  Exon 1 in mitochondrial gene  *21S rDNA*,  which has 2 exons and 1 intron |
| 21S-R2 | TAAAGCTGCATAGGGTCTTT |
| 21S-F3 | GTAAGCTATGTTTGCCACCT | [21S1 (Exon 2)]  Exon 2 in mitochondrial gene  *21S rDNA*,  which has 2 exons and 1 intron |
| 21S-R3 | AATTGACGAACAGTCAAACC |
| ACT1-F1 | TTGGATTCCGGTGATGGTGT | [ACT1-1]  mRNA gene *ACT1*, which is transcribed by RNA Pol II |
| ACT1-R1 | CGGCCAAATCGATTCTCAAA |
| ADH1-F1 | CGGTATCAAATGGTTGAACG | [ADH1-1]  mRNA gene *ADH1*, which is transcribed by RNA Pol II |
| ADH1-R1 | ACAAGTCAGCGTGAGGACAG |
| CYH2-F1 | CCTTTCATCTGTATCCCGTA | [Promoter-Exon 1]  Region encompassing promoter and Exon 1 of *RPL28* (*CYH2*), which is transcribed by RNA  Pol II |
| CYH2-R1 | TTAGTGAATCTGGAAGGCAT |
| CYH2-F3 | GTTGAGACGGCTTATTTGAG | [Intron]  Intron region of mRNA gene *RPL28* (*CYH2*), which is transcribed by RNA Pol II |
| CYH2-R3 | AGGCTATGCACACTTTCTTT |
| CYH2-F4 | CACCACAGAATTAACATGGA | [Exon 2]  Exon 2 region of mRNA gene *RPL28* (*CYH2*), which is transcribed by RNA Pol II |
| CYH2-R4 | GAAATGAGCTTGTTGCTTGT |
| PMA1-3’end-F | TGACAGATTGATGAACGGTA | [PMA1]  mRNA gene *PMA1*, which is transcribed by RNA Pol II |
| PMA1-3’end-R | CGTGTTGAGTAGAGACTCTTTG |
| CEN16-F1 | TGAGCAAACAATTTGAACAG | [CEN16]  Centromere of chromosome XVI |
| CEN16-R1 | CCGATTTCGCTTTAGAAC |
| Tel01-F | ACGATCATTTGTTAGCGTTT | [Tel01L]  Telomere 1L |
| Tel01-R | CTCCAATTACCCATATCCAA |
| **B: Standard PCR primers** |  |  |
| **Name** | **Sequence^a,b,c^** | **specifications** |
| TYB OUT | GAACATTGCTGATGTGATGACA | Primer pair used for analysis of integration of TY1 elements at  tRNA^GLY^ sites |
| SUF16 | GGCAACGTTGGATTTTACCAC |
| **C: ChIP-seq primers** |  |  |
| **Name** | **Sequence^a^** | **specifications** |
| Adapter *T-Solexa | ACACTCTTTCCCTACACGACGCTCTTCCGATC*T | Standard PE adapters  * indicates a Phosphorothioate link, [Phos] indicates a 5’-phosphorylated primer |
| (Phos)Adapter-solexa | [Phos]GATCGGAAGAGCGGTTCAGCAGGAATGCCGAG |
| Primer 1.1 | **CAAGCAGAAGACGGCATACGAGATCGGTCTCGGCATTCCTGCTGAACCGCTCTTCCGATC*T** | Solexa Library PE PCR primers  * indicates a Phosphorothioate link |
| Primer 2.1 | **AATGATACGGCGACCACCGAGATCTACACTCTTTCCCTACACGACGCTCTTCCGATC*T** |

**^a^** Oligonucleotides are indicated (5’-3’)

**^b^** Nyswaner KM, Checkley MA, Yi M, Stephens RM, Garfinkel DJ (2008) Chromatin-associated genes protect the yeast genome from Ty1 insertional mutagenesis. Genetics 178: 197-214.

**^c^** Scholes DT, Banerjee M, Bowen B, Curcio MJ (2001) Multiple regulators of Ty1 transposition in Saccharomyces cerevisiae have conserved roles in genome maintenance. Genetics 159: 1449-1465.
