# Supplementary material for: Genome-Wide Distribution of RNA-DNA Hybrids Identifies RNase H Targets in tRNA Genes, Retrotransposons and Mitochondria
Source: PLoS Genet. 2014 Oct 30;10(10):e1004716. doi: 10.1371/journal.pgen.1004716 (PMC4214602; doi:10.1371/journal.pgen.1004716)
Supplement: Protocol S1 — Bioinformatic analysis. (DOC) [file pgen.1004716.s020.doc]

**Protocol S1: Bioinformatic Analysis**

Perl, bash shell and R scripts that were used along with their associated data files can be accessed from the following site: <https://github.com/AlastairKerr/ElHage2014>.

**A- Gene profile plots**

Regions of interest were normalized for length by dividing each in to a set of equally sized windows:

**A-i)** Heatmaps for tRNA genes show discrete values for each window in each gene.

gene= 50 windows. Flanks= 1000 bases upstream and downstream/ 100 windows each.

**A-ii)** Average profile plots were constructed either by box plotting the values in each window or by calculating the median read depth at each window across all regions of interest.

*# Intronless (e-genes)* gene plots*:* gene= 100 windows.

*## intron-containing (i-genes)* gene plots*:* Exon1= 50 windows. Intron1= 50 windows. Exon 2= 50 windows. Exon 1 <100 bases were extended upstream to 100 bases in length. The AUG start codon is defined as the 5’end of Exon 1 in these plots.

*### Ty1 elements plots:* gene= 200 windows. Ty1 elements in strain BY4741 (S288c) are as annotated in the SacCer3 genome ([http://www.yeastgenome.org](http://www.yeastgenome.org/)).

**B- mRNA expression**

Raw transcriptome sequencing (RNA-seq) reads of exponentially growing BY4741 cells (see [101] and GEO accession number GSM617028) were aligned to the sacCer3 genome with Tophat v2.09. Protein-coding genes were placed in to four categories of expression based on the number of RNA-seq reads per base of exon (see Fig. S10).

**C- NET-seq**

Raw Native elongating transcript sequencing (NET-seq) reads of exponentially growing BY4741 cells (see [101] and GEO accession number GSM617027) were aligned to the sacCer3 genome with Novoalign v2.07 and the number of NET-seq reads per base of exon were generated for protein-coding genes (see Fig. S10).

**D- Thermodynamic profiles of polynucleotide sequences**

A non-overlapping window of 9 bases was examined along the genome. In each window two values were calculated: the concentrations of [G+C] nucleotides and the thermodynamic properties of RNA/DNA [designated (pre-mRNA)/DNA in the manuscript] or DNA/DNA sequences (see Figs. S14 and S16). [G+C] is simply the ratio of G or C in the 9 bases window. The thermodynamic properties represent the free energy required to unwind polynucleotide sequences with defined length as described in [12]. Basically, the higher the helical stability (note that sites rich with rG:dC have the highest helical stability), the higher the free energy cost (ΔG) for strand separation. Conversely, the lower the helical stability the lower the free energy cost (ΔG) for strand separation. A window of 9 bases was chosen to reflect the number of bases in the transcription bubble as described in [12].
